# LAIR-1 agonism as a therapy for acute myeloid leukemia

**DOI:** 10.1172/JCI169519

**Published:** 2023-11-15

**Authors:** Rustin R. Lovewell, Junshik Hong, Subhadip Kundu, Carly M. Fielder, Qianni Hu, Kwang Woon Kim, Haley E. Ramsey, Agnieszka E. Gorska, Londa S. Fuller, Linjie Tian, Priyanka Kothari, Ana Paucarmayta, Emily F. Mason, Ingrid Meza, Yanira Manzanarez, Jason Bosiacki, Karla Maloveste, Ngan Mitchell, Emilia A. Barbu, Aaron Morawski, Sebastien Maloveste, Zac Cusumano, Shashank J. Patel, Michael R. Savona, Solomon Langermann, Han Myint, Dallas B. Flies, Tae Kon Kim

**Affiliations:** 1 NextCure Inc., Beltsville, Maryland, USA.; 2 Division of Hematology/Oncology, Department of Medicine, Vanderbilt University Medical Center, Nashville, Tennessee, USA.; 3 Seoul National University Hospital and; 4 Department of Internal Medicine, Seoul National University College of Medicine, Seoul, South Korea.; 5 Department of Pathology, Microbiology and Immunology, Vanderbilt University Medical Center,; 6 Vanderbilt Center for Immunobiology,; 7 Vanderbilt-Ingram Cancer Center, and; 8 Program in Cancer Biology, Vanderbilt University, Nashville, Tennessee, USA.; 9 Veterans Affairs Tennessee Valley Healthcare, Nashville, Tennessee, USA.

**Keywords:** Oncology, Leukemias

## Abstract

Effective eradication of leukemic stem cells (LSCs) remains the greatest challenge in treating acute myeloid leukemia (AML). The immune receptor LAIR-1 has been shown to regulate LSC survival; however, the therapeutic potential of this pathway remains unexplored. We developed a therapeutic LAIR-1 agonist antibody, NC525, that induced cell death of LSCs, but not healthy hematopoietic stem cells in vitro, and killed LSCs and AML blasts in both cell- and patient-derived xenograft models. We showed that LAIR-1 agonism drives a unique apoptotic signaling program in leukemic cells that was enhanced in the presence of collagen. NC525 also significantly improved the activity of azacitidine and venetoclax to establish LAIR-1 targeting as a therapeutic strategy for AML that may synergize with standard-of-care therapies.

## Graphical abstract

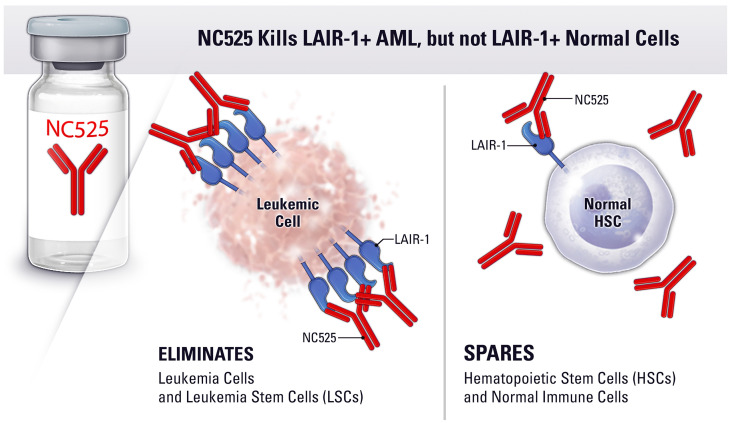

## Introduction

Acute leukemias are characterized by the uncontrolled production of malignant hematopoietic progenitors. Acute myeloid leukemia (AML) is the most common adult acute leukemia (1). While extensive research has led to the recent approval of additional therapies for AML (2), there remains a significant unmet need for patients who fail to respond to or relapse after standard-of-care (SoC) treatments. This failure is predominantly due to the resistance of leukemic stem cells (LSCs) to SoC (3). LSCs are self-renewing leukemia-initiating cells at the apex of the hierarchy of leukemia cells in the bone marrow (BM). LSCs give rise to daughter leukemic blasts, initiate disease when transplanted to immunodeficient animals, and propagate upon serial transplantation (4). The self-renewal capacity of LSCs leads to refractoriness to initial treatment or relapse in patients who achieved remission after initial treatment (5), making LSCs a critical target for next-generation therapeutics against AML.

Meyaard et al. first described leukocyte-associated immunoglobulin-like receptor-1 (LAIR-1), a collagen-binding immunoreceptor tyrosine-based inhibitory motif–bearing (ITIM-bearing) inhibitory receptor that can recruit Src homology region 2 domain–containing phosphatase-1 (SHP-1; PTPN6) and C-terminal Src kinase (CSK) (6–9). LAIR-1 is restricted to the hematopoietic compartment, particularly myeloid cells, but also T cells, B cells, and NK cells (10). When collagenous ligands bind to LAIR-1, receptor clustering results in the phosphorylation of LAIR-1 ITIM domains, which then recruit and phosphorylate SHP-1 to trigger downstream immune-inhibitory signaling (10). Like other inhibitory immune receptors, the function of LAIR-1 in healthy cells is to dampen immune responses in non-homeostatic or disease environments (11–13).

Two early studies demonstrated a role for LAIR-1–mediated inhibition of leukemic cells in AML. One study showed that LAIR-1 ligation on leukemia cells inhibited IκBα activation to prevent nuclear factor-κB (NF-κB) translocation into the nucleus, resulting in programmed cell death (14). A follow-up study showed that receptor clustering blocked the proliferation of AML blasts and led to subsequent cellular apoptosis, an effect dependent on LAIR-1 ITIM signal transduction through SHP-1 (15).

A later study found that LAIR-1 has been linked to cell stemness and disease development in leukemia (16). In this study, LAIR-1 on AML cells led to downstream signaling through Ca^++^/calmodulin-dependent protein kinase I (CAMK1) and cAMP response element–binding protein (CREB), which was implicated in sustaining AML stem cell activity. While LAIR-1 was dispensable for normal hematopoiesis, knockdown of LAIR-1 in human leukemia cells increased apoptosis in vitro and reduced AML development in murine models.

Here, we show that enhanced LAIR-1 signaling induced by an agonist monoclonal antibody (mAb) drives a unique apoptotic program in AML cells compared with healthy immune cells. We uncover a mechanism of control over AML growth, with the LAIR-1 receptor acting as a central regulator determining AML fate decisions between cancer cell growth tolerance or suppression. As such, these studies provide a new understanding of LAIR-1 biology, and a novel therapeutic approach, to the best of our knowledge, to treat AML by targeting LAIR-1 with an agonist mAb.

## Results

### LAIR-1 is a target for AML therapy.

**Figure 1:**
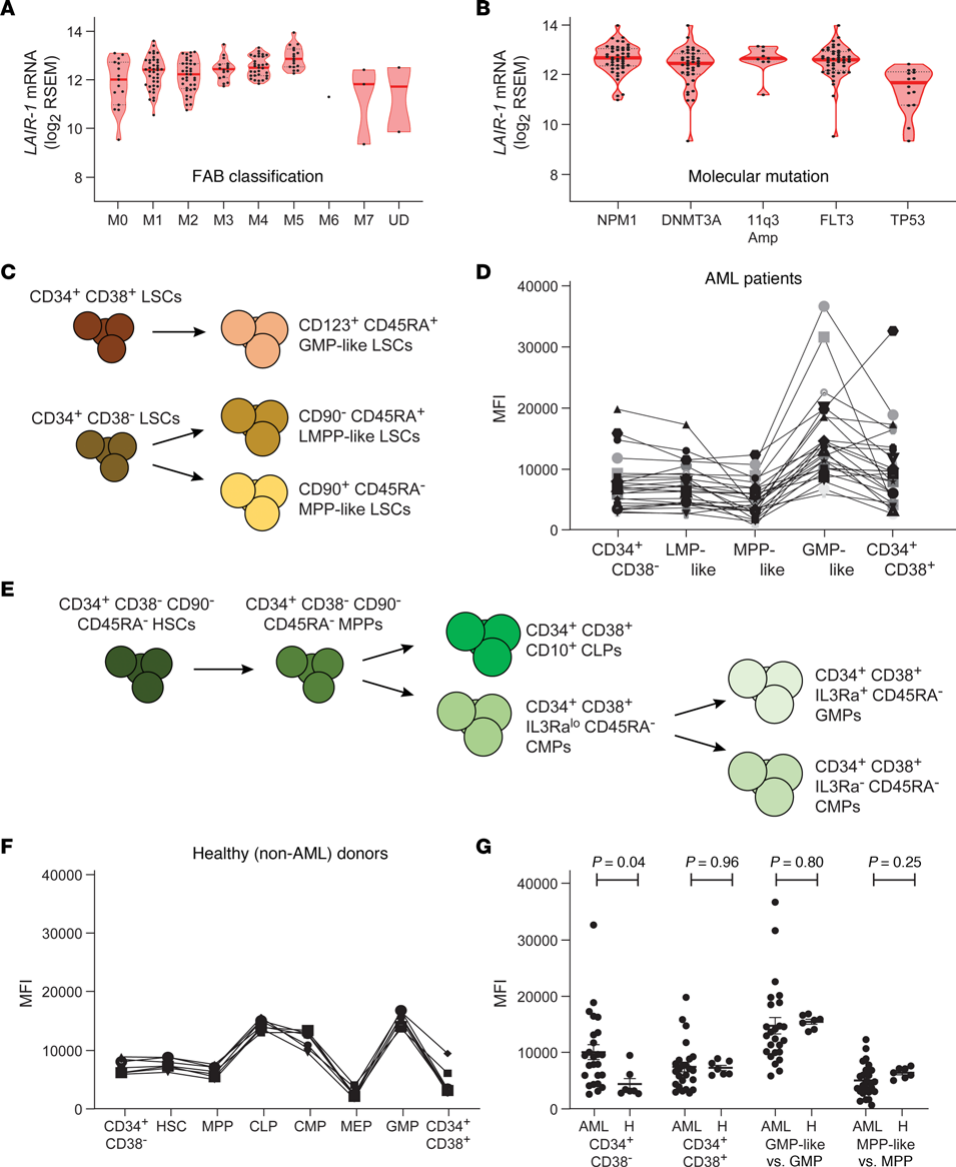
LAIR-1 is a target for AML therapy. (**A** and **B**) LAIR-1 transcript levels, as measured by RNA sequencing and quantified by RNA Sequencing by Expectation and Maximization (RSEM) software, in AML patient samples clustered by French-American-British (FAB) classification (**A**) or by molecular mutation (**B**). UD, undiagnosed (non-AML). *n* = 1–47 patient samples per group. (**C**) Illustration of leukemopoiesis from LSC precursors into granulocyte-macrophage progenitor–like (GMP-like) LSCs, lymphoid-primed multipotent progenitor–like (LMPP-like) LSCs, or multipotential progenitor–like (MPP-like) LSCs. (**D**) Mean fluorescence intensity (MFI) of LAIR-1 cell surface expression on the indicated LSC subpopulations. Each symbol/line represents a unique patient. *n* = 25 AML patients. (**E**) Illustration of normal hematopoiesis from hematopoietic stem cells (HSCs) into multipotential progenitors (MPPs), common lymphoid progenitors (CLPs), common myeloid progenitors (CMPs), or granulocyte-monocyte progenitors (GMPs). (**F**) MFI of LAIR-1 cell surface expression on the indicated HSC subpopulations. Each symbol/line represents a unique donor. *n* = 7 non-AML healthy donors. (**G**) Comparison of LAIR-1 cell surface expression on cells from comparable compartments from AML patients or healthy donors (H). Each dot represents a unique donor/patient. *n* = 7 healthy donors or 25 AML patients. *P* value determined by Student’s *t* test. Data are shown as the mean ± SEM.

Studies have reported aberrantly elevated LAIR-1 expression on leukemic cells (16, 17). To extend this finding, we assessed the expression of LAIR-1 on human AML cells (Supplemental Table 1; supplemental material available online with this article; https://doi.org/10.1172/JCI169519DS1). The Cancer Genome Atlas was analyzed for *LAIR1* mRNA levels from AML patients representing each disease subtype as described by the French-American-British (FAB) classification system (M0–M7), and likewise for expression of *LAIR1* in patients with AML-associated mutations. We observed no difference in *LAIR1* expression levels between various subtypes, with each subtype except M7 having higher mean expression than undiagnosed donors (Figure 1A). Similarly, *LAIR1* mRNA levels did not correlate with any mutation (Figure 1B). Because leukemic blasts in the peripheral blood arise from a pool of self-renewing LSCs within the BM, we determined cell surface expression of LAIR-1 on different lineage subsets of human AML cells (4, 18) (Figure 1C) using flow cytometry (Supplemental Figure 1). LAIR-1 levels were variable across AML patients, with granulocyte-macrophage progenitor–like (GMP-like) and CD34^+^CD38^+^ subsets expressing the highest overall levels of LAIR-1 receptor (Figure 1D). Healthy donor hematopoietic stem progenitor cell (HSPC) subsets (Figure 1E) displayed little variability (Figure 1F). Direct comparisons of similar subsets from AML or healthy donors from Figure 1, D and F, illustrated more broad variability among AML patients (Figure 1G).

### LAIR-1 agonism inhibits the growth of LSCs but not healthy hematopoietic stem cells.

To test the effect of LAIR-1 engagement on LSCs, we used the LAIR-1 agonist mAb NC525. NC525 is a humanized mAb with a functional IgG1 that specifically binds to human LAIR-1 but does not bind to mouse LAIR-1 protein (Supplemental Figure 2, A and B). NC525 blocks collagen:LAIR-1 interactions (Supplemental Figure 2, C and D). However, NC525 can also induce human LAIR-1 signaling (agonist) upon engagement as indicated by phosphorylation of SHP-1 (Supplemental Figure 2, E and F). NC525 also promotes antibody-dependent cellular cytotoxicity of AML target cells (Supplemental Figure 2G).

**Figure 2:**
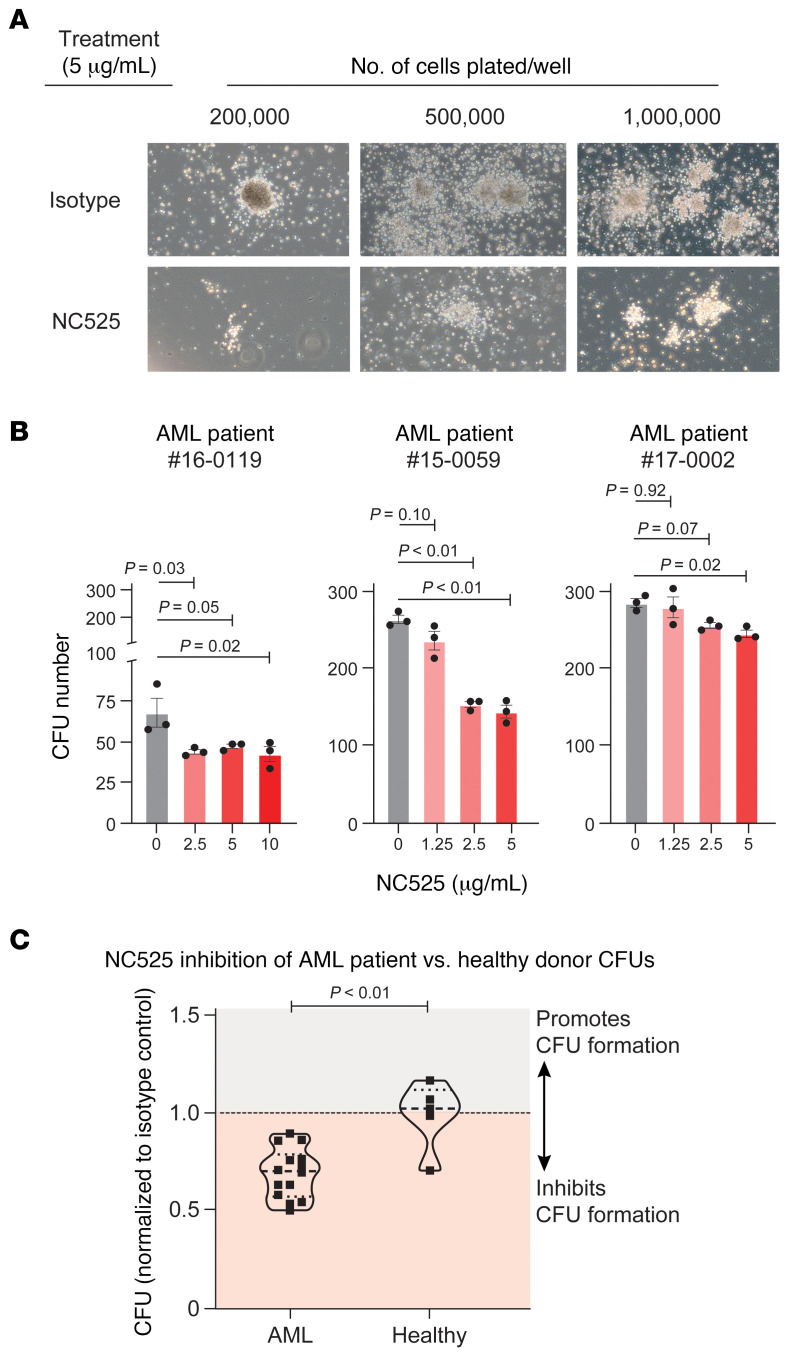
LAIR-1 agonist mAb NC525 inhibits colony formation in LSCs but not healthy stem cells. (**A**) Representative images of ex vivo colony formation of LSCs from AML patient 19-0029 when plated at the indicated cell number and treated with 5 μg /mL of NC525 or isotype control. (**B**) Colony-forming units (CFUs) formed by ex vivo plating of (left to right) 50,000, 20,000, or 12,000 LSCs per well from the indicated AML patient during titrated treatment with anti–LAIR-1 agonist mAb NC525. *n* = 3 technical replicates per group. *P* values determined by 1-way ANOVA with multiple comparisons. (**C**) CFU formation from healthy donor BM or AML patient BM treated with 5 μg /mL NC525. Values normalized to isotype control. *n* = 14 AML biological replicates or 5 healthy biological replicates. *P* value determined by Student’s *t* test.

To determine whether LAIR-1 engagement by NC525 impacts LSCs, we performed ex vivo colony-forming unit (CFU) assays with AML BM recovered from multiple patients (Supplemental Table 2). Colony formation of AML BM cells identifies leukemic cells that are broadly defined as progenitors of leukemic blasts, and thus colony formation can be used to quantify LSCs (19). Compared with isotype or vehicle controls, LAIR-1 engagement by NC525 significantly decreased CFU formation in LSCs in a dose-dependent manner (Figure 2, A and B). However, no change in CFU formation was observed with NC525 treatment of BM cells from healthy control donors (Figure 2C), suggesting that LAIR-1 regulates self-renewal more strongly in LSCs.

### LAIR-1 engagement eradicates primary and secondary AML in patient-derived xenograft models.

**Figure 3:**
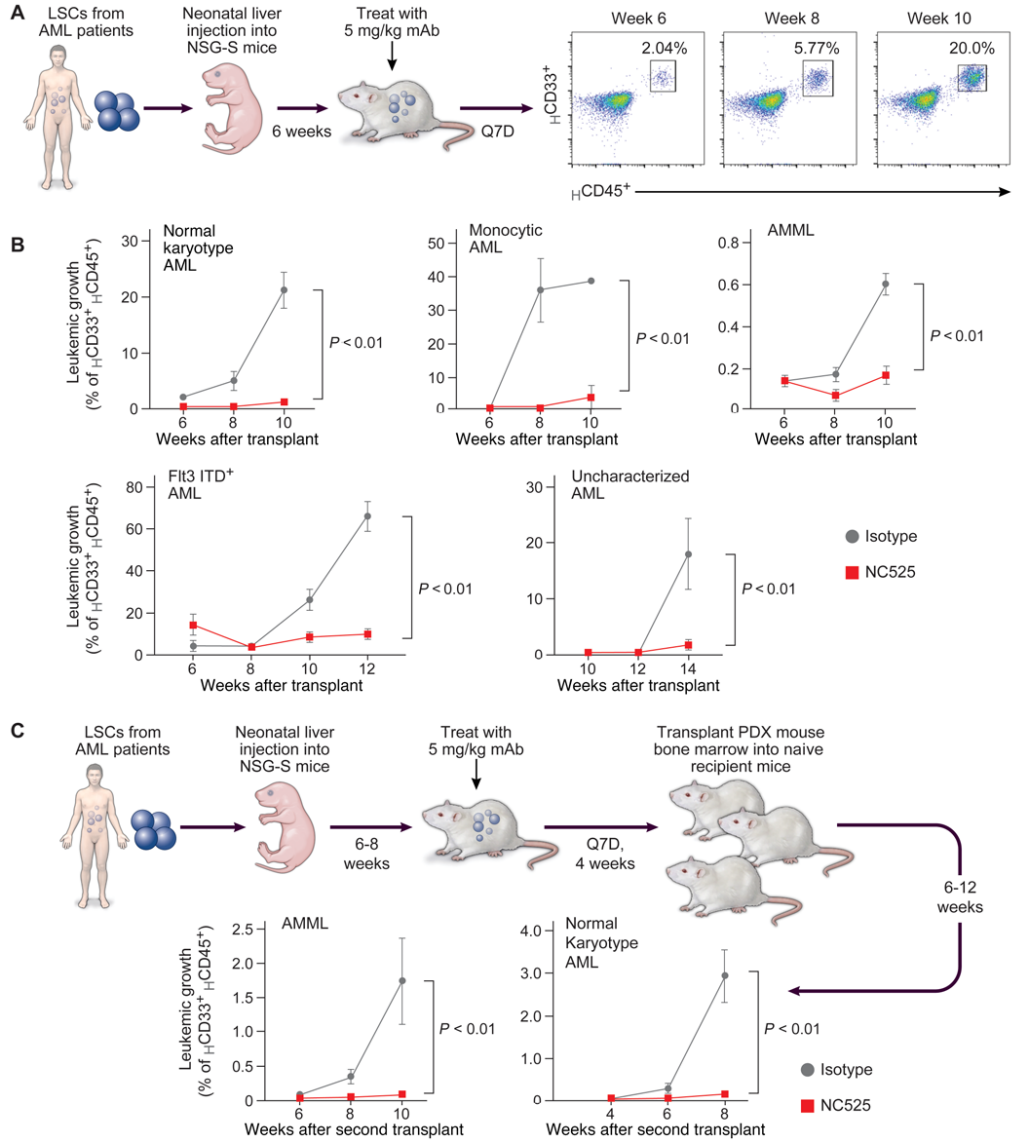
LAIR-1 engagement eradicates primary and secondary AML in patient-derived xenograft models. (**A**) Schematic of the AML patient–derived xenograft (PDX) model and representative scatterplots of human (H) CD33^+^CD45^+^ leukemic cells in circulation at the indicated time after engraftment. Subgated population represents percent of parent. (**B**) Leukemic growth, as measured by the percentage of circulating _H_CD33^+^_H_CD45^+^ cells, in PDX mice engrafted with BM from donors with normal-karyotype AML (*n* = 4–5 mice per group), monocytic AML (*n* = 4–5 mice per group), acute myelomonocytic leukemia (AMML) (*n* = 4 mice per group), FLT3-ITD^+^ AML (*n* = 8 mice per group), or uncharacterized AML (*n* = 5 mice per group). Engrafted mice were treated with 5 mg/kg isotype control (gray) or NC525 (red). (**C**) Schematic of PDX secondary transplant model, where BM from PDX mice engrafted and treated as above was harvested and secondarily transplanted into naive recipient mice. Graphs show leukemic growth in secondary recipient mice after receiving BM from AMML PDX animals or normal-karyotype AML PDX animals that had been treated with 5 mg/kg isotype control (gray) or NC525 (red). *n* = 3 mice per group. *P* values calculated by 2-way ANOVA. Data are shown as the mean ± SEM.

To test the in vivo impact of LAIR-1 engagement on LSCs, we performed AML patient–derived xenograft (PDX) modeling. LSCs from AML patients were engrafted into non-lethally irradiated neonatal mice, and human cell proliferation was subsequently measured by quantification of the percentage of circulating leukemic cells (Figure 3A). PDX mice treated with NC525 did not develop disease, with less than 10% human CD45^+^CD33^+^ cells in circulation at any time point, while control mice had up to 70% leukemic cells in circulation by 12 weeks after engraftment (Figure 3B). NC525-mediated AML suppression was observed across multiple donors and AML subtypes, including normal-karyotype AML, monocytic AML, acute myelomonocytic leukemia (AMML), FLT3-ITD^+^ AML, and uncharacterized AML (Figure 3B). To delineate whether the in vivo suppressive effect was due to removal of circulating blasts or eradication of LSCs in the BM, we performed secondary transplant experiments from PDX donor mice that had been engrafted with AML BM cells (either AMML or normal karyotype). Mouse BM harvested from PDX animals that had been treated for 4 weeks with either NC525 or isotype control was transplanted into tumor-naive mice (secondary transplant) (Figure 3C). No further treatment was given after the secondary transplant. Mice that received BM from isotype control–treated donors developed AML, while those that received BM from NC525-treated animals showed no propagation of leukemia, indicating that LAIR-1 engagement by NC525 eradicated LSCs within the BM of the PDX donor animals (Figure 3C).

### NC525 agonism of LAIR-1 induces AML cell death.

**Figure 4:**
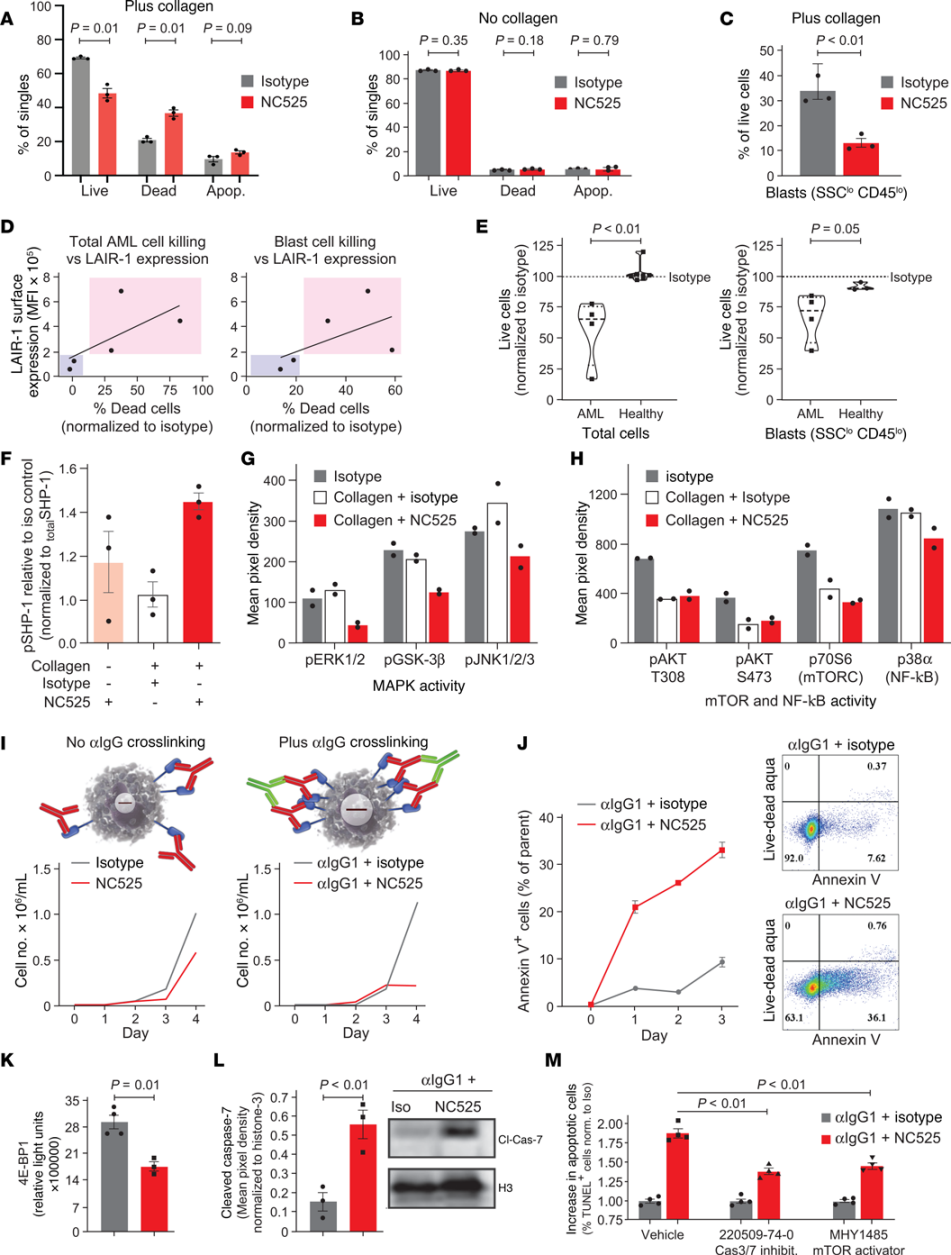
NC525 agonism of LAIR-1 induces AML cell death. (**A**–**C**) Blood leukocytes after ex vivo treatment with 10 μg/mL isotype (gray) or NC525 (red) with (**A**) or without (**B**) exogenous collagen or CD45^lo^SSC^lo^ blasts with collagen (**C**). *n* = 3 technical replicates. (**D**) Percentage dead leukocytes or blasts treated as in **A** and **B** relative to LAIR-1 surface expression. Each dot represents an individual patient. Red or blue shading indicates LAIR-1 greater or less than 20,000 arbitrary units, respectively. Line represents linear regression. (**E**) Total live cells or blasts from healthy or AML donors after treatment as indicated in **A** and **B**. *n* = 4–7 donors. (**F**) AlphaLISA of phosphorylated SHP-1 relative to total SHP-1 in AML PBMCs, normalized to isotype treatment. *n* = 3 technical replicates. (**G** and **H**) Duplicate dot blots from phospho-arrays of AML PBMCs quantified for MAPK (**G**) or mTOR and NF-κB activity (**H**). (**I**) Schematic of LAIR-1 (blue) clustering using anti-IgG (green) and NC525 (red). In vitro growth of MV4-11-LAIR-1^overexpressing^ cells treated with isotype (gray) or NC525 (red) in the absence or presence of anti-IgG. (**J**) Annexin V staining of cells treated as in **I**, with representative scatterplot of apoptotic annexin V^+^ Live-Dead Aqua^–^ cells at day 3 of culture. (**K** and **L**) 4E-BP1 expression as measured by Lumit assay (**K**) or cleaved caspase-7 as measured by Western blot (**L**) of cells treated as shown in **I**. Mean pixel density normalized to histone-3 for triplicate samples. (**M**) Percentage TUNEL^+^ MV4-11-LAIR-1^overexpressing^ cells at day 3 of treatment plus DMSO vehicle, 50 μM 220509-74-0 (caspase-3/7 inhibitor), or 50 μM MHY1485 (mTOR activator). Values normalized to isotype for each respective condition. *n* = 3–4 technical replicates. *P* values calculated by Student’s *t* test. Error bars represent SEM.

To define the mechanism of LAIR-1–mediated leukemic growth arrest and cell death, we quantified the degree of cell death induced through LAIR-1 ligation by performing ex vivo culture of red blood cell–depleted (RBC-depleted) AML patient whole blood in the presence of NC525 or isotype control, followed by measurement of live and dead cell populations by flow cytometry (Supplemental Table 3 and Supplemental Figure 3A). In support of in vivo data, LAIR-1 engagement significantly increased cell death and decreased the CD45^lo^ side scatter–low (SSC^lo^) blast population (Supplemental Figure 3, B and C). NC525-mediated cell death was enhanced in the presence of plate-coated collagen (Figure 4, A–C). This intriguing finding suggested that LAIR-1 signaling in coordination with other collagen binding molecules directs leukemic cell fate. Indeed, some studies have suggested that AML cells can undergo collagen-dependent reprogramming within the BM (20). In addition, the ability of NC525 to induce leukemic cell death in the presence of collagen was dependent on the level of LAIR-1 expression (Figure 4D). Notably, LAIR-1 engagement by NC525 did not kill blood leukocytes from healthy (non-AML) donors (Figure 4E). This result was similar to observations of hematopoietic stem cell (HSC) CFU formation during NC525 treatment (Figure 2C) and indicates that NC525 induces differential signaling in AML cells versus healthy cells despite potentially similar expression levels.

Because SHP-1 has been reported as the major adaptor molecule associated with LAIR-1 signal transduction (15), we treated primary patient AML blasts with NC525 in the presence or absence of collagen and measured the phosphorylation status of SHP-1 by AlphaLISA. NC525 increased phosphorylation by approximately 25% compared with collagen, and cotreatment of cells with NC525 and collagen elicited an additive effect, increasing phosphorylation levels by approximately 50% (Figure 4F). To further extrapolate the LAIR-1 signaling axis, we probed phospho-immunoreceptors and downstream kinase molecules on primary AML cells using dot blot arrays. Quantification of downstream intracellular phospho-activity during NC525 and collagen treatment revealed a pattern of decreased phosphorylation in protein species including ERK1/2, GSK-3β, and JNK (Figure 4G and Supplemental Figure 4A). Moreover, LAIR-1 agonism by NC525 in the context of collagen decreased the activation of AKT, mTORC, and NF-κB (Figure 4H and Supplemental Figure 4A). These findings suggested that NC525 suppressed survival pathways including the mTOR self-renewal pathway in AML cells (21) to block proliferation and induce cell death. To determine whether this signaling paradigm was specific to leukemic cells, we performed reverse-phase protein microarray analysis on healthy donor or AML donor PBMCs treated with NC525 in the presence of collagen. Data showed that NC525 significantly suppressed mTOR activity and activated caspase-7 in AML donor cells but not healthy cells (Supplemental Figure 5, A and B). These collective data illustrate a differential pattern of signaling specific to AML cells that is induced by NC525, which is enhanced in the presence of collagen.

Our data and previous studies suggested differential LAIR-1 signaling dynamics within AML cells (14, 15). We hypothesized that a major role of collagen in LAIR-1–mediated cell death is to promote the accumulation of localized LAIR-1 receptors capable of overcoming a signaling threshold that dictates leukemic cell fate. As such, strong LAIR-1 clustering, even in the absence of a collagen matrix, would overcome this threshold to inhibit cell growth. To test this, an MV4-11-LAIR-1^overexpressing^ cell line was used. LAIR-1 clustering on the cell surface was induced by culturing of cells with NC525 plus an anti–human IgG antibody that binds to the Fc domain of the NC525 mAb (Figure 4I). Growth of LAIR-1^overexpressing^ cells cultured with NC525 alone was only moderately suppressed compared with isotype control (Figure 4I, left). However, proliferation of cells cultured under clustering conditions was inhibited entirely after 3 days of culture (Figure 4I, right), with a commensurate increase in annexin V^+^ cells (Figure 4J). To further test induction of cell death by NC525-mediated LAIR-1 signaling, mTOR and caspase-3/7 assays on anti-IgG–clustered cells were performed. We observed significant suppression of the mTORC1 target protein 4E-BP1 (Figure 4K) as well as a substantial increase in activated caspase-7 (Figure 4L) in the respective NC525 treatment groups. Moreover, NC525-induced apoptosis of AML cells could be partially but significantly reversed by addition of a small-molecule activator of mTOR or a small-molecule inhibitor of caspase-3/7 (Figure 4M). Meanwhile, NC525 clustering of LAIR-1 in healthy CD34^+^ cells induced minimal changes in signaling activity (Supplemental Figure 5C). These data further support that NC525 clustering of LAIR-1 induces signaling pathways specific to leukemic cells that inhibit growth processes and promote cell death.

Collectively, these results define a mechanism of control over AML growth, with the LAIR-1 receptor acting as a central regulator determining AML fate decisions between cancer cell growth tolerance or suppression.

### NC525-mediated AML killing in vivo is dependent on LAIR-1 expression level.

**Figure 5:**
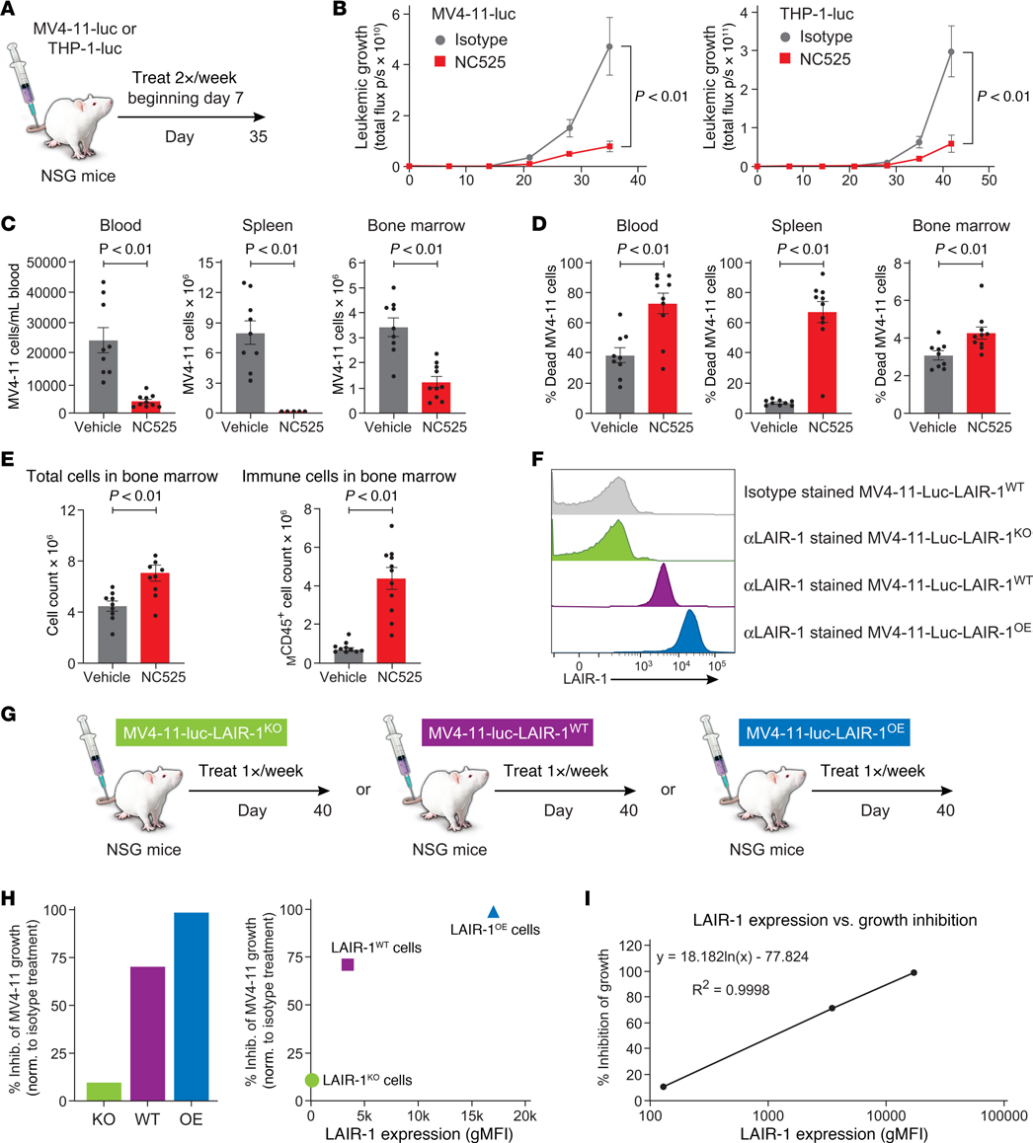
NC525-mediated AML killing in vivo is dependent on LAIR-1 expression level. (**A**) Schematic of the MV4-11–luciferase or THP-1–luciferase cell-derived xenograft (CDX) model of AML. (**B**) In vivo leukemic growth as measured by whole-body luminescence of MV4-11–luciferase cells (left) or THP-1–luciferase cells (right) in CDX mice treated with 10 mg/kg isotype control (gray) or NC525 (red). *n* = 8 mice per group. *P* values determined by 2-way ANOVA. (**C** and **D**) MV4-11 cell counts (**C**) or percent dead MV4-11 cells (**D**) in the blood, spleen, or BM of CDX mice treated with vehicle control (gray) or 10 μg/mL NC525 (red). (**E**) Total cell counts or mouse (M) CD45^+^ cell counts in the BM of CDX mice treated with vehicle control (gray) or 10 μg/mL NC525 (red). *n* = 9–10 mice per group. *P* values determined by Student’s *t* test. Data are shown as the mean ± SEM. (**F**) Representative histograms of LAIR-1 cell surface expression on the indicated cell lines. (**G**) Schematic of CDX model systems to test the inhibition of leukemic growth as a function of LAIR-1 expression. (**H**) Percent inhibition of MV4-11-LAIR-1–knockout (green), MV4-11-LAIR-1–wild-type (purple), or MV4-11-LAIR-1–overexpression cell growth in vivo (normalized to the mean of the respective isotype controls) after treatment with 10 mg/kg NC525 (left) and plotted against LAIR-1 geometric mean fluorescence intensity (gMFI) (right). (**I**) Percent inhibition of MV4-11 growth fit to a logarithmic regression curve of LAIR-1 expression. *n* = 9 mice per group.

To evaluate the in vivo dynamics of LAIR-1–mediated suppression of leukemic cell growth, we used a cell-derived xenograft (CDX) model of AML allowing for mechanistic studies that cannot be readily performed in PDX models. We first validated LAIR-1 expression on several AML cell lines (Supplemental Table 4), finding that most AML cell lines express LAIR-1 (Supplemental Figure 6). MV4-11 and THP-1 cells were selected for CDX modeling based on established protocols (22, 23), where naive NSG mice that lack T or B cells were engrafted with human MV4-11 cells or THP-1 cells that had been transduced to constitutively express RedFluc luciferase reporter (PerkinElmer) (Figure 5A). Within these models, NC525 engages human LAIR-1 on engrafted leukemic cells but does not recognize endogenously expressed murine LAIR-1 (Supplemental Figure 2, A and B). As in the PDX studies, NC525 inhibited leukemic cell growth in both the MV4-11 CDX model (Figure 5B, left) and the THP-1 model (Figure 5B, right).

Further evaluation using the MV4-11 model showed that AML cells were nearly absent in the blood, spleen, and BM of NC525-treated mice (Figure 5C). Concomitantly, the percentage of dead MV4-11 cells was increased in blood, spleen, and BM (Figure 5D), supporting the hypothesis that LAIR-1 engagement by NC525 actively induced cell death of circulating and tissue-resident AML cells in vivo. Notably, the inhibition of AML growth in the BM allowed healthy mouse immune cells to be retained (Figure 5E).

To further evaluate the effect of NC525 treatment, BM from the MV4-11 model was harvested on day 27 to analyze changes in RNA expression using a NanoString metabolic gene expression panel. To do so, human CD45^+^ cells were isolated ex vivo from BM to remove mouse cells before NanoString nCounter analysis. Twelve genes were significantly upregulated and 28 genes were significantly downregulated in AML cells during NC525 treatment (Supplemental Figure 7 and Supplemental Table 5). Of those downregulated genes, we observed that mTOR, SOS1, and BCL-2 were downregulated 2.3-fold, 2.3-fold, and 1.8-fold, respectively (Supplemental Table 5).

The correlation of LAIR-1 expression level with NC525 activity observed in vitro was also evaluated in vivo using the MV4-11 model. MV4-11 LAIR-1–knockout (KO) cells were compared with LAIR-1–overexpressing (OE) cells and wild-type (WT) cells (Figure 5, F and G). Results showed that NC525 more strongly inhibited LAIR-1–OE MV4-11 growth compared with LAIR-1–WT (Figure 5H). Inhibition of growth fit a logarithmic curve of LAIR-1 expression *y* = 18.182ln(*x*) – 77.824, as measured by mean fluorescence intensity (Figure 5I).

The MV4-11 CDX model provides a powerful tool to elucidate cell-intrinsic effects of LAIR-1 signal transduction in leukemic cells, and the activity of NC525 in this model indicates that the mechanism of leukemic cell removal is not dependent on T cells or adaptive immunity given that NSG mice do not have an intact human immune compartment. However, we also evaluated the effect of NC525 in the presence of healthy human immune cell populations in vivo. NSG mice that were reconstituted for 11–16 weeks with healthy human CD34^+^ stem cells were treated for 5 weeks with NC525 to evaluate changes in human immune cell populations (Supplemental Figure 8A). Results showed that NC525 had minimal effects on healthy human immune cells in the spleen or the BM (Supplemental Figure 8B).

To evaluate the effects of NC525 on healthy human lymphocytes within the context of AML, the same MV4-11 model was used, but with engraftment of healthy human PBMCs. Suppression of leukemic growth by NC525 was similar to, or better than, that in the non-PBMC-engrafted CDX models (Supplemental Figure 8C), indicating that human T cells did not alter NC525 antileukemic activity. Furthermore, we found no discernible change in total human CD3^+^ T cells, CD4^+^ T cells, CD8^+^ T cells, CD20^+^ B cells, or CD25 expression on T cells recovered from the spleen (Supplemental Figure 8D) or BM (Supplemental Figure 8E) after NC525 treatment.

### LAIR-1 signaling restricts AML survival pathways in vivo.

**Figure 6:**
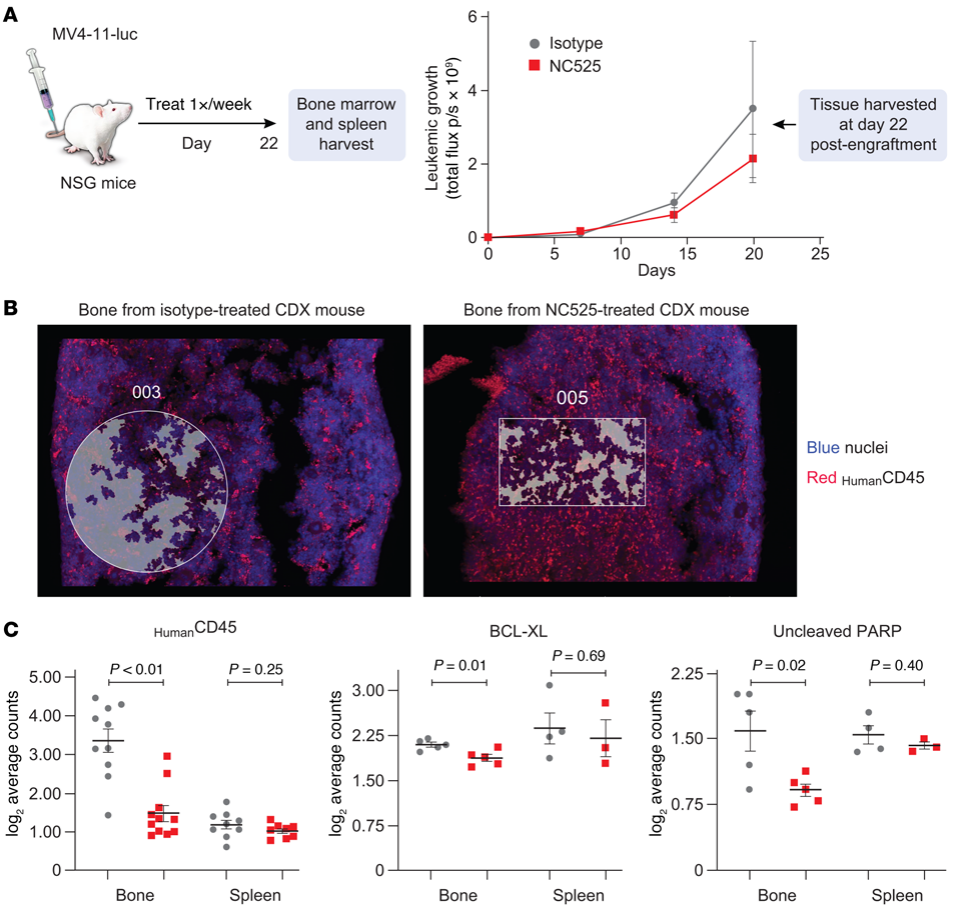
LAIR-1 signaling restricts AML survival signaling pathways in vivo. (**A**) Model schematic (left) and MV4-11-luciferase growth (right) in CDX mice used for digital spatial imaging and target protein quantification. *n* = 6 mice per group. (**B**) Representative images of CDX mouse bones stained with DAPI (blue) and anti–human CD45 (red) used to quantify the number of MV4-11 cells in the indicated region of interest (ROI) (highlighted). ROI area is equal between samples. (**C**) Protein reads of human CD45, BCL-XL, or uncleaved PARP from bone or spleen harvested from CDX mice at day 22 after engraftment. Read counts are normalized to histone H3 and ribosomal protein S6. *n* = 3–11 quantified tissue regions across 2 isotype-treated or 2 NC525-treated mice. Data are shown as the mean ± SEM. *P* values determined by Student’s *t* test.

While quantification of the phosphorylation status of cell survival molecules is helpful in gaining insight into downstream signaling dynamics, the method is limited to measuring a single posttranslational modification. It does not capture non-phosphate-mediated signal transduction events that may be important in the leukemic cell fate axis centered around LAIR-1 engagement. NanoString digital spatial profiling (DSP) coupled with protein expression panel analysis was performed on mouse BM and splenic tissue from NC525-treated MV4-11 CDX mice to address this. To capture temporally dependent cellular changes, murine tissues were harvested at the initiation of MV4-11 growth divergence between treatment groups (Figure 6A). At this time point, NC525 did not elicit any difference in MV4-11 tissue localization (Figure 6B). However, in support of in vivo CDX disease profiling (Figure 5B), LAIR-1 engagement by NC525 did cause a reduction in AML cells (represented by human CD45^+^ cells) in the bones of CDX mice (Figure 6C, left). Protein panel expression analysis showed that AML cells displayed decreased levels of anti-apoptotic BCL-XL (Figure 6C, middle) and uncleaved PARP (Figure 6C, right) in bones but not in spleens of mice treated with NC525. No significant changes were observed in other proteins in the tested panels.

### NC525 synergizes with AML standard-of-care therapy.

A combination regimen of VEN/AZA, consisting of venetoclax (VEN), which blocks anti-apoptotic B cell lymphoma-2 (Bcl-2) protein, and azacytidine (AZA), which inhibits DNA methyltransferase, has become a standard of care (SoC) for treatment of elderly patient AML. Two of the AML whole-blood samples that responded to ex vivo NC525 treatment were from patients on VEN therapy, and 3 of the samples were from patients previously treated with hypomethylating agents (Figure 4D and Supplemental Table 3), supporting the hypothesis that NC525 induces AML cell apoptosis in SoC-treated patient populations. One reason that AML patients become resistant to VEN/AZA is the upregulation of BCL-XL. Because we observed significant reduction in BCL-XL during NC525 treatment, we tested the activity of NC525 with VEN/AZA using ex vivo assays and CDX and PDX in vivo models.

**Figure 7:**
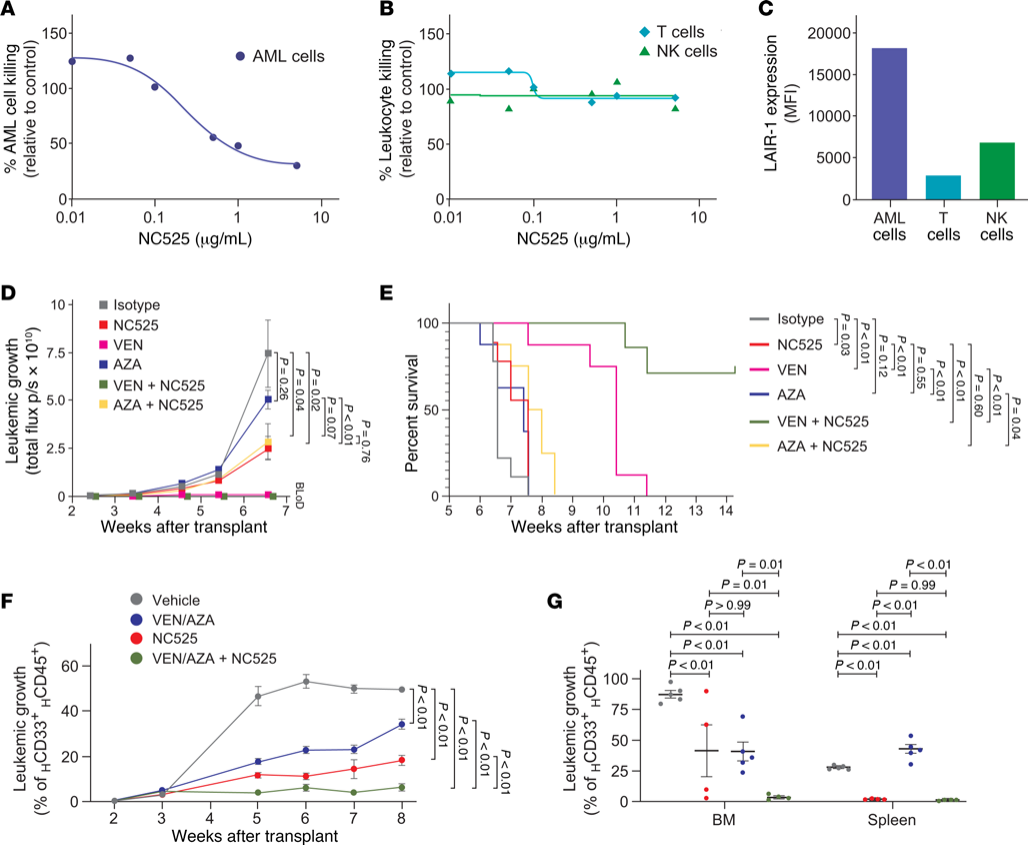
NC525 synergizes with AML standard-of-care therapy. Ex vivo killing of VEN/AZA–treated AML patient leukemic cells (**A**) or T cells or NK cells (**B**). Values normalized to vehicle control. Lines represent linear regression of NC525 concentration versus normalized cell killing. (**C**) LAIR-1 surface expression of VEN/AZA–treated AML patient leukemic cells, T cells, or NK cells. (**D** and **E**) In vivo leukemic growth as measured by whole-body luminescence of MV4-11–luciferase cells (**D**) or survival (**E**) of CDX mice treated with 10 mg/kg isotype control (gray) or NC525 (red) or 100 mg/kg VEN (pink) or 0.5 mg/kg AZA (blue) or combination therapy (green or yellow, respectively). BLoD, below limit of detection. *n* = 9 mice per group. *P* values determined by 2-way ANOVA or log-rank (Mantel-Cox) test, respectively. (**F** and **G**) Leukemic growth in the blood (**F**) and in the spleen and BM (**G**) at week 8 after transplant of AML PDX mice treated with vehicle (gray), VEN/AZA (blue), NC525 (red), or VEN/AZA plus NC525 (green). *n* = 5–10 mice per group. AML cells in spleen and BM compared from 4–5 mice per group. Data are shown as the mean ± SEM. *P* values determined by 2-way ANOVA or 1-way ANOVA with multiple comparisons.

In fresh BM leukemic cells from a patient treated with VEN/AZA, we observed dose-dependent killing of SoC-resistant AML cells by NC525, with up to 70% killing observed at 5 μg/mL of antibody (Figure 7A). Notably, no impact on healthy T cells or NK cells was observed (Figure 7B), although BM AML cells had 4-fold higher LAIR-1 expression compared with patient-matched T cells or NK cells (Figure 7C). Next, the MV4-11 model was used to evaluate NC525 activity in comparison with, and in combination with, AZA or VEN treatments. NC525 monotherapy had significantly better activity than AZA monotherapy at physiologically relevant doses, and AZA cotreatment with NC525 did not inhibit NC525 activity (Figure 7D). While the physiologically relevant regimen of VEN monotherapy suppressed MV4-11 in vivo growth below the limit of detection within the time frame of the growth curve, survival of mice after challenge was significantly increased in animals treated with the combination of NC525 and VEN over either monotherapy alone (Figure 7E). To further delineate the potential of NC525 against SoC-resistant AML, PDX modeling using engrafted BM from an additional VEN/AZA–resistant patient was performed. NC525 treatment significantly reduced AML disease as measured by circulating blast cell burden (Figure 7F) and AML cells in the spleen and BM (Figure 7G). Combining NC525 with VEN/AZA further reduced AML disease, suggesting synergistic activity (Figure 7, F and G). These results show not only that LAIR-1 is a viable target for therapeutic intervention of AML, but that the NC525 agonist antibody can work in concert with current clinical therapeutics to eradicate disease and improve patient outcomes.

**Figure 8:**
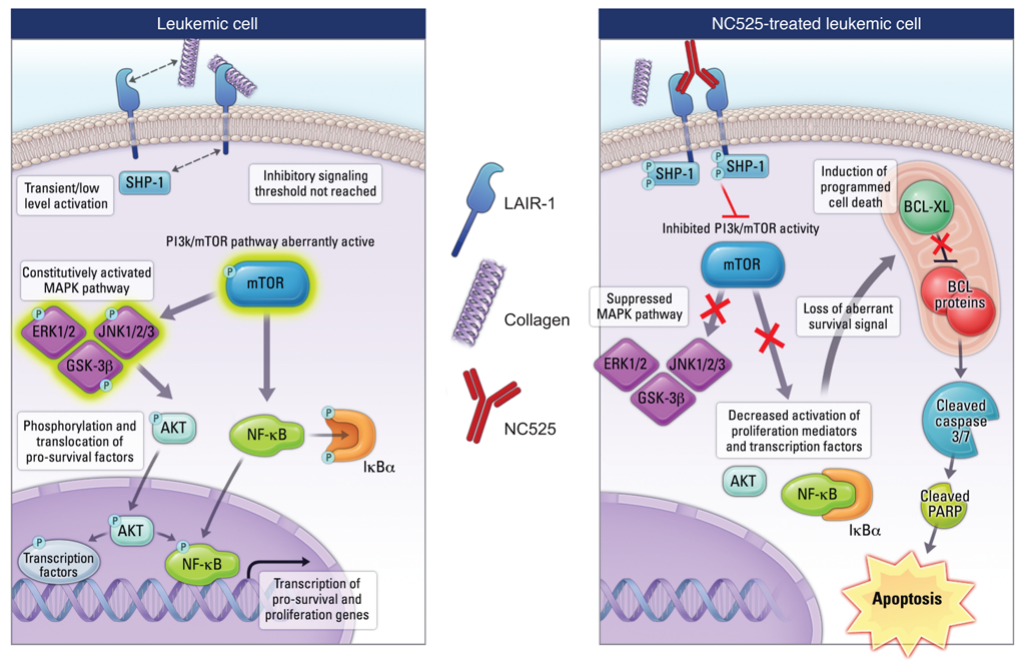
NC525 induces AML apoptosis through enhanced LAIR-1 clustering and SHP-1 phosphorylation. Schematic of LAIR-1–induced cell death in leukemic cells. Engagement of LAIR-1 on AML cells by agonist mAb NC525 induces enhanced SHP-1 phosphorylation that blocks aberrant PI3K/mTOR activity, leading to the suppression of constitutively active MAPK signaling and the self-renewal mechanisms promoted by AKT and NF-κB. This loss of proliferative signaling induces the deactivation of BCL-XL, which releases an apoptotic cascade through caspase-7 and PARP, culminating in programmed cell death.

Overall, these collective results indicate that heightened LAIR-1 signaling via NC525 disrupts receptor pathways that are critical to leukemia cell survival and triggers suppressive pathways that lead to apoptosis and leukemic cell death (Figure 8). Agonist targeting of LAIR-1 is thus a unique and promising strategy for AML therapeutic intervention.

## Discussion

This study provides evidence that targeting LAIR-1^+^ leukemic cells with a LAIR-1 agonist mAb is an effective approach for inhibiting and killing AML blasts and LSCs while sparing normal HSCs. Specifically, these experiments demonstrate that engagement of LAIR-1 by the agonist mAb NC525 induces cell death in leukemic blasts and LSCs to sustain efficacy and reduce relapse, as demonstrated in a secondary transplant PDX model. NC525 also robustly inhibits leukemia in two different CDX models and several PDX models. Importantly, NC525 kills VEN/AZA–resistant AML cells in vitro and in vivo, and combinatorial treatment with VEN/AZA elicits an additive therapeutic effect.

Salvage therapies, including VEN monotherapy for relapsed/refractory AML, have minimal effect. Recent approvals of VEN-based therapies for the treatment of newly diagnosed AML in adults 75 years or older have changed the landscape of the therapeutic paradigm (24). However, further challenges emerge for those who relapse or are refractory after front-line VEN-based therapies. Maiti et al. initially showed that the median overall survival after VEN-based therapy was 2.4 months, with an objective response rate (ORR) of 21% (25), while compiled VEN-based studies have confirmed poor survival after VEN failure (26). VEN-based salvage therapy after allogeneic transplant achieved 38% ORR with a modest progression-free survival among the responders (27). Therefore, there is an urgent need for novel therapies to abrogate resistance to VEN-based therapies.

NC525 may fulfill the need for a therapeutic that overcomes resistance to VEN-based therapies. Our data show a significant downregulation of BCL-XL during NC525 treatment. A combination of VEN/AZA with NC525 may effectively suppress BCL-2/MCL-1 and downregulate BCL-XL to induce synergistic killing of AML cells. As follow-up translational studies, it will be critical to test the efficacy of NC525 in additional leukemia models, including VEN/AZA nonresponder models and de novo versus relapsed/refractory leukemia, and in further combination studies.

VEN-based therapies, like currently available and experimental therapies, are associated with significant severe cytopenias that drive serious and fatal complications such as neutropenic fever, sepsis, fungal infections, and bleeding. This study shows that NC525 may provide an effective therapeutic intervention in AML with minimal effects on healthy hematopoietic cells or leukocytes. Therapeutically, the effective removal of LSCs and leukemia-initiating cells may prevent relapses that are extremely common with currently available AML therapies.

The finding that NC525 does not trigger suppression of mTOR or activation of caspase-7 in healthy cells, as it does in AML cells, may relate to how pathways downstream of LAIR-1 drive leukemic pathogenesis. One potential scenario that could explain the unique effect of NC525 on AML cells is that LAIR-1 signaling in healthy cells is not apoptotic at homoeostatic levels of mTOR activity, but when aberrant mTOR/AKT/NF-κB activity is driving leukemic self-renewal, the loss of proliferation signal elicited through LAIR-1 cross-linking permits apoptotic self-elimination mechanisms to execute programmed cell death. However, our data also suggest that mTOR is not the only pathway being altered by LAIR-1 signaling, and further dissection of the distinct signaling pathways altered by LAIR-1 signaling and NC525 will be the focus of future studies.

The remarkable finding that leukemic cell death induction is enhanced by a collagen matrix in the presence of NC525, even when NC525 blocks collagen binding to LAIR-1, speaks to the complexity of collagen-mediated signaling. Since the initial identification of LAIR-1 as a receptor for collagen, it has been suggested that receptor clustering is critical for LAIR-1 signal transduction (7, 28). However, in the absence of collagen-mediated LAIR-1 clustering, enhanced clustering of LAIR-1 by NC525 with an anti-IgG mAb resulted in LAIR-1 signaling that overcame the collagen-mediated clustering requirement. Therefore, a threshold level of LAIR-1 signal appears essential for the induction of AML cell death, and a threshold level of signal transduction may not be met in healthy cells where LAIR-1 functions to regulate immune processes primarily under non-homeostatic conditions (11–13).

LAIR-1 is not the sole immunoreceptor that engages collagenous ligands. Our data suggest that collagen matrices act as an information network that educates cell membranes to cluster LAIR-1 and other collagen-binding receptors. Discoidin domain receptors 1 and 2 (DDR1 and DDR2), α_1_β_1_ integrin, α_2_β_1_ integrin, and others can also exhibit immunomodulation upon collagen binding, which illustrates the complex signaling dynamics dictated by ECM proteins and their receptors (29). This extended network likely integrates LAIR-1 signaling induced by NC525 agonism. Importantly, LAIR-1 is the only known collagen receptor with inhibitory signaling capacity, thus further implicating a critical role for LAIR-1–mediated signaling in AML cells (30, 31). Understanding this network of receptors and regulation by LAIR-1 signaling will be the focus of future research studies.

It has been well established that collagen and the ECM regulate various cell membrane dynamics (32) and that ECM ligands can impact signaling and subsequent cell fate of leukemic cells, particularly in the BM (20, 33). These differential signaling dynamics could be the reason for the discrepancy between reports showing LAIR-1 activation of cell survival mechanisms, such as CAMK1/CREB (16, 34), and reports showing LAIR-1–mediated growth arrest and cell death (14, 15). Compellingly, we also detected increased CAMK1/CREB activity in AML cells under LAIR-1 agonism (Supplemental Figure 4A), supporting data published by Kang et al. (16). However, in our assays, the increase did not promote increased cell survival. This disconnect may indicate that CAMK1/CREB is dispensable for AML cell survival in the context of collagen matrices, where the LAIR-1 signaling network suppresses alternative downstream mediators, such as NF-κB, MAPK, and Src family kinases, to the point that the signaling threshold overcomes prosurvival mechanisms to instead result in programmed cell death.

NC525 contains a functional Fc IgG1 domain that can mediate antibody-dependent cellular cytotoxicity (ADCC) of target cells. However, given the lack of activity on healthy immune cells, the primary mechanism by which NC525 promotes cell death appears to be through LAIR-1 signaling, which may be enhanced by interactions with Fc receptors. Beyond potential interactions in *trans*, intracellular signaling through Fc receptors has been shown to impact cell survival in many contexts, including leukemia (35). Additional studies will be performed to elucidate the roles of signaling versus ADCC in leukemic cells.

In summary, this study points to LAIR-1 as a central inhibitory receptor on leukemic cells that can be targeted therapeutically with an agonist antibody to inhibit growth and induce leukemic cell death. An open-label, non-randomized, phase I study to determine the safety and tolerability of NC525, and to assess the clinical benefit in subjects with advanced myeloid neoplasms, is ongoing.

## Methods

### Patient samples.

All patients provided informed consent to participate in this study, which had the approval and guidance of the Institutional Review Board at Vanderbilt University Medical Center. Human BM aspirate samples were coded and processed by the Hematologic Malignancies Tissue Repository at Vanderbilt University Medical Center. For ex vivo studies, AML patient or healthy donor whole blood was purchased through StemExpress, Fred Hutchinson Cancer Center (Seattle, Washington, USA), or Yale Cooperative Center of Excellence in Hematology (New Haven, Connecticut, USA). Whole blood was collected into EDTA Vacutainer tubes (BD), kept chilled, and tested within 24 hours of draw. All samples were deidentified.

### Animals.

All mouse procedures were performed in accordance with institutional guidelines at Vanderbilt University. Mice were maintained according to NIH animal care guidelines, and experimental protocols described in this study were approved by Vanderbilt University’s Institutional Animal Care and Use Committees. NSG (NOD-scid-IL2Rγ^null^) and NSG-SGM3 [NOD.Cg-PrkdcscidIl2rgtm1WjlTg(CMV-IL3,CSF2,KITLG)1Eav/MloySzJ] mice were purchased from The Jackson Laboratory and housed in a specific pathogen–free environment for the duration of the study.

### Flow cytometry.

All human cell preparations were more than 95% viable by trypan blue exclusion. 1 × 10^6^ thawed or fresh BM mononuclear cells were stained with Zombie Aqua viability dye (BioLegend) and annexin V–Alexa Fluor 647. Human cells were identified by FITC-conjugated BD FastImmune Anti-Human Lineage Cocktail 1 (lin 1) (CD3, CD14, CD16, CD19, CD20, CD56) (BD Biosciences, catalog 340546). Specific human cell surface markers were identified in PDX studies using antibodies against human CD45–APC (BioLegend, catalog 304012) and CD33-PE (BioLegend, catalog 303403); in healthy CD34^+^ stem cell engraftment models using antibodies against CD3–PerCP/Cy5.5 (BioLegend, catalog 344808), CD4-FITC (BioLegend, catalog 300538), CD8–APC/eFluor 780 (eBioscience, catalog 47-0087-42), CD20-PE (BioLegend, catalog 302306), CD45-PacBlue (BioLegend, catalog 368540), and CD25-BV711 (BioLegend, catalog 302636); in CDX models using antibodies against mouse CD45–PE (BioLegend, catalog 157604) and human CD45–PacBlue (BioLegend, catalog 368540); in in vitro killing assays using antibodies against CD45-BV711 (BioLegend, catalog 304050), CD20-PE (BioLegend, catalog 302306), and CD3–PerCP/Cy5.5 (BioLegend, catalog 344808); and in BM phenotyping assays using antibodies against CD34–PE/Cy7 (BD Biosciences, catalog 34811), CD38-BV785 (BioLegend, catalog 303530), CD90-BV421 (BioLegend, catalog 328122), CD45RA–APC/Cy7 (BioLegend, catalog 304128), CD117 (BioLegend, catalog 313213), and LAIR-1–Alexa Fluor 647 (BD Biosciences, catalog 565326). After staining, cells were washed, resuspended in PBS with 1%–4% paraformaldehyde, quantified with a Celesta flow cytometer (BD Biosciences) or an Attune NXT flow cytometer (Thermo Fisher Scientific), and analyzed using FlowJo software (Tree Star).

For most of the analyses, at least 3 × 10^5^ total events were analyzed, with sequential gating of BM mononuclear cells. For CD34^+^ AML cells, lineage-negative cells were demarcated as CD34^+^CD38^–^CD45RA^–^CD90^–^ (multipotential progenitor–like [MPP-like] LSCs), CD34^+^CD38^–^CD45RA^+^CD90^–^ (lymphoid-primed MPP–like [LMPP-like] LSCs), and CD34^+^CD38^+^CD45RA^+^CD123^+^ (GMP-like LSCs). For CD34^–^ AML cells, lineage-negative cells were demarcated as CD34^–^CD117^+^ (GM precursor–like LSCs) (4). LAIR-1 expression was assessed in each LSC subset compared with isotype control. For healthy donor BM, the same mAb conjugates were used except CD127–PerCP/Cy5.5 (BioLegend, catalog 351322) was substituted for CD117–PerCP/Cy5.5 in the AML panel. Healthy donor BM cells were gated according to the recommendations by Pang et al. (36). HSPCs were demarcated as CD34^+^CD38^–^CD45RA^–^CD90^+^ (HSCs), CD34^+^CD38^–^CD45RA^–^CD90^–^ (MPPs), CD34^+^CD38^+^CD127^+^ (common lymphoid progenitors), CD34^+^CD38^+^CD45RA^–^CD123^+^ (common myeloid progenitors), CD34^+^CD38^+^CD45RA^–^CD123^–^ (MEPs, and CD34^+^CD38^+^CD45RA^+^CD123^+^ (GMPs). LAIR-1 expression was assessed on each HSPC subset compared with isotype control. In both AML and healthy donor BM cell analyses, fluorescence minus one was used to separate CD45RA^+^ from CD45RA^–^, CD90^+^ from CD90^–^, CD123^+^ from CD123^–^, and CD127^+^ from CD127^–^ populations.

MV4-11-LAIR-1^KO^ and MV4-11^WT^ cells were used to validate the specificity of LAIR-1 mAbs. AML cell lines MV4-11, THP-1, HL-60, and U937 were purchased from ATCC; Kasumi1, NB4, HEL1 (gifts from Manoj Pillai, Yale University, New Haven, Connecticut, USA), and MOLM14 (gift from Martin Carroll, University of Pennsylvania, Philadelphia, Pennsylvania, USA) were used as described.

### TCGA analysis.

Normalized RNA Sequencing by Expectation and Maximization (RSEM) mRNA expression data on 162 samples from AML patients included in The Cancer Genome Atlas (TCGA) project were downloaded from cBioPortal (www.cbioportal.org; hosted by Memorial Sloan Kettering Cancer Center) together with information on corresponding clinical, mutational, and cytogenetic parameters.

### Antibody cell binding and reporter cell assays.

For cell binding assays, 5 × 10^4^ UT-140 cells or mouse LAIR-1–transfected 293T cells (ATCC) per well were plated in 200 μL PBS in a 96-well round-bottom plate, centrifuged for 4 minutes at 500*g* for a single wash, then blocked for 10 minutes on ice with flow cytometry buffer (PBS plus 2% FBS plus 0.1 mM EDTA) containing 1:50 dilution of TruStain Fc block (BioLegend). Cells were subsequently stained for 30 minutes on ice with titrated concentrations of soluble Alexa Fluor 647–labeled NC525 or isotype control. Cells were then washed 3 times as previously described, and binding was measured by an Attune NXT flow cytometer. For reporter cell assays, flat-bottom 96-well tissue culture plates were coated with 10 μg/mL per well of the indicated antibody in PBS or 2 μg /mL of ligand in 0.01N HCl overnight at 4°C. Wells were washed once with 200 μL sterile PBS. 5 × 10^4^ to 1 × 10^5^ cells per well were plated in 100 μL complete RPMI medium (cRPMI) containing titrated concentrations of NC525 or isotype control, then incubated overnight at 37°C. The next day, cells were transferred to a 96-well round-bottom plate, centrifuged for 4 minutes at 500*g*, and resuspended in 200 μL of flow cytometry buffer. Cells were then centrifuged and resuspended for a total of 3 washes. GFP expression was measured by flow cytometry using an Attune NXT flow cytometer.

### Colony-forming unit assay.

Cryopreserved AML BM cells or healthy donor CD34^+^ cells were thawed and plated in 96-well plates. Cells were engaged with the indicated concentrations of NC525 or isotype control for 30 minutes at room temperature. Subsequently, cells were diluted in IMDM with 2% FBS and mixed with semisolid methylcellulose-based medium (MethoCult H4435 Enriched, StemCell Technologies), which contains human cytokines (stem cell factor, IL-3, IL-6, EPO, G-CSF, GM-CSF). Then, between 1 × 10^4^ and 2.5 × 10^5^ LSCs or between 1 × 10^3^ and 5 × 10^3^ healthy donor CD34^+^ cells per well were plated on 6-well SmartDishes (StemCell Technologies). The cell-loaded plates were incubated at 37°C with 5% CO_2_ in air and at least 95% humidity. On day 14, colony formation was counted by STEMvision (StemCell Technologies), an automated and standardized colony counting instrument. The automated results were confirmed and edited manually by the STEMvision Colony Marker software.

### Patient-derived xenograft models.

Newborn (3–5 days from birth) NSG-SGM3 pups were sublethally (200 cGy) irradiated. Twelve hours after irradiation, mice were intrahepatically transplanted with 0.2 × 10^6^ AML BM cells or normal CD34^+^ HSCs from cryopreserved stocks. Six weeks after transplant, blood was collected to assess engraftment of progenitor cells via flow cytometry, staining for human CD45, human CD33, and human CD3. Once engraftment of human cells was confirmed, 5 mg/kg of anti–human LAIR-1 mAb NC525 or isotype control was injected intraperitoneally (i.p.) starting at week 6 and continuing weekly up to a total of 4 doses. At the indicated time points after treatment, blood was collected to assess in vivo AML proliferation as a measure of NC525 antileukemic activity as compared with isotype control. AML cell proliferation in recipient mice was assessed by flow cytometry by quantification of the percentage of human CD33^+^ human CD45^+^ cells in peripheral blood.

For secondary transplant studies, mice were engrafted as above. BM from treated mice was transplanted into sublethally irradiated NSG-SGM3 recipient mice. No treatment was provided to recipient mice. At the indicated time points after treatment, blood was collected to assess the proliferation of AML cells as a measure of NC525 treatment effect on LSCs as compared with isotype control.

For the PDX SoC combination study, adult NSG-SGM3 mice (8 weeks old) were engrafted with 2 × 10^6^ BM cells from 1 AML patient, then treated with 20 mg/kg venetoclax (5 days on and 2 days off) for 5 weeks and 1.5 mg/kg azacytidine every other day for the first week only. NC525 was administered i.p. once per week for 5 weeks in combination with venetoclax and azacytidine. Blood was harvested as described above to measure in vivo AML proliferation, and spleens and BM were harvested at endpoint.

For assessment of mAb impacts on normal human immune cells, NSG-SGM3 CD34^+^ fully engrafted humanized mice (>25% human CD45^+^ leukocytes in circulation) were purchased from The Jackson Laboratory (stock 2523). Mice were received approximately 12 weeks after engraftment, and the experiment was initiated after 1 week of acclimatization. Mice were treated i.p. with 5 mg/kg mAb weekly for 4 weeks. One week after the final mAb dose, mice were euthanized, and splenocytes, lymph nodes, and BM cells were harvested for total cell counts, followed by analysis of CD45^+^, CD3^+^, CD4^+^, CD8^+^, CD14^+^, CD11b^+^, CD20^+^, and CD56^+^ cell percentages. Absolute numbers of cell subpopulations were calculated, as well as percentages of each population subset as a percentage of total CD45^+^ cells and total leukocytes.

### Cell-derived xenograft models.

NSG mice were engrafted with 2 × 10^6^ THP-1–luciferase cells or 2 × 10^6^ MV4-11–luciferase cells via tail vein injection. In vivo AML proliferation was assessed using weekly bioluminescence in vivo imaging system (IVIS) imaging, starting on day 7 after challenge. Treatment with 10 mg/kg mAb was performed by i.p. injection starting on day 8 and continuing once or twice a week until the end of the study. For PBMC-engrafted CDX studies, 1 × 10^7^ human PBMCs were engrafted into NSG mice via tail vein injection 1 day before MV4-11 challenge as described above. For the SoC CDX study, mice were challenged and treated as above, with cohorts that included 100 mg/kg venetoclax administered via oral gavage or 0.5 mg/kg azacytidine administered i.p. for 5 days on and 2 days off over 4 weeks.

### Coculture and ex vivo killing assays.

For the ADCC assay, MV4-11 cells were labeled with DELFIA BATDA reagent (PerkinElmer) at 4 μL/1 × 10^6^ cells for 30 minutes at 37°C, washed twice, then resuspended in cRPMI with freshly thawed PBMCs at an effector/target ratio of 80:1 (0.8 × 10^6^ PBMCs to 1 × 10^4^ MV4-11 cells). Cells were cocultured with titrated concentrations of soluble NC525 or isotype for 2 hours, then treated with DELFIA Eu-solution and quantified on an ENVISION plate reader (PerkinElmer). Percent cell lysis was calculated by [(experimental release counts – spontaneous release counts)/(maximum release counts – background counts)] × 100.

For ex vivo killing assays, fresh whole blood was RBC-depleted using the EasySep RBC Depletion Reagent (StemCell Technologies) following the manufacturer’s protocol. Cell number was determined by VI-Cell XR cell counter. 2 × 10^5^ cells per well were plated into 96-well plates lacking or containing 50 μg/mL precoated human collagen I (StemCell Technologies) in 200 μL of cRPMI containing 10 μg/mL of soluble NC525 or isotype. Plates were centrifuged at 100*g* for 2 minutes, then incubated for 20 hours at 37°C. For BM killing assays, fresh BM cells were cultured in IMDM containing 4 mM l-glutamine, 10% autologous plasma, and titrated soluble NC525 for 24 hours at 37°C. At the end of the incubation period, cells were transferred to a 96-well round-bottom plate for cell staining and flow cytometry as described above.

### Western blot, AlphaLISA, and signaling arrays.

For phospho-arrays of AML patient PBMCs, 6-well plates were left uncoated or precoated with 100 μg/mL of collagen I (StemCell Technologies). Coated plates were washed 3 times with sterile PBS, and then 10 μg/mL of IgG1 isotype or NC525 was coated for 1 hour at 37°C. Plates were washed once with sterile PBS. 1.5 × 10^6^ thawed AML patient PBMCs or CD34^+^ enriched cord blood cells were added to each well and cultured for 2 hours. Cells were then lysed using kit-provided lysis buffer, and dot blots were performed using the Proteome Profiler Human Phospho-Kinase Array Kit (R&D Systems, ARY003C) or the Proteome Profiler Human Phospho-Immunoreceptor Array Kit (R&D Systems, ARY004B) following the manufacturer’s protocol.

For reverse-phase protein microarray (RPPA) analysis, healthy or AML PBMCs were cultured as described above, and lysates were printed onto nitrocellulose-backed slides in triplicate. Slides were treated with ReBlot (MilliporeSigma), washed twice in DPBS (Gibco), placed into blocking reagent (I-Block, Applied Biosystems), and then stained using the DAKO GenPoint kit with a DAKO Autostainer Plus. Slides were incubated with primary antibodies for 30 minutes at room temperature and then incubated with secondary antibody. Protein detection was amplified via horseradish peroxidase–mediated biotinyl tyramide deposition and visualized using a fluorescent probe (LI-COR). For total protein determination, a nitrocellulose slide was treated with 1% sodium hydroxide and incubated in a destain solution (7% acetic acid, 30% methanol), and a 0.2% Fast Green solution was applied. Images of stained RPPA and Fast Green–stained slides were captured on an InnoScan 710-AL (Innopsys) and analyzed using Mapix software.

For assessment of SHP-1, human PBMCs or monocytes isolated from PBMCs using a StemCell monocyte isolation kit (StemCell Technologies) were seeded at 2 × 10^6^ cells per well of a 12-well plate.

Cells were stimulated with 20 ng/mL C1q (CompTech) or 100 μg/mL collagen I (StemCell Technologies) with or without 10 μg/mL NC525 or isotype control. The plate was centrifuged at 350*g* for 2 minutes. After a 5-minute incubation at 37°C, cells were pelleted, lysed in the presence of phosphatase inhibitors, and either processed for Western blotting as described below or quantified by AlphaLISA following the manufacturer’s instructions (PerkinElmer, alsu-pshp1-b-hv).

### In vitro clustering assay.

1 × 10^6^ MV-411-LAIR-1^WT^ or MV-411-LAIR-1^overexpressing^ cells per well were seeded in 6-well plates. Cells were subsequently treated with 10 μg/mL soluble IgG1 isotype (NextCure, NP782) or NC525. Clustering was induced by the addition of 7.5 μg/mL anti–human IgG1 (R&D Systems, MAB9894), and cell growth was quantified over 5 days by flow cytometry.

For Lumit assay, MV-411-LAIR-1^overexpressing^ cells were cultured and treated as above in serum-free media (StemSpan Leukemic Cell Culture Kit, 09605, 02691) for 4 days. Cells were then directly lysed in wells via the manufacturer-supplied lysis buffer and assayed according to the manufacturer’s protocol (Promega, Lumit Immunoassay Cellular System W1331).

For TUNEL assays, 5 × 10^4^ MV-411-LAIR-1^overexpressing^ cells were treated as above for 3 days in 96-well plates in the presence of 10 μM caspase-3/7 inhibitor (MilliporeSigma, 218826), 10 μM mTOR (MilliporeSigma, SML0810) activator, or DMSO vehicle control. Apoptosis was measured daily by TUNEL staining following the manufacturer’s protocol (Thermo Fisher Scientific, A23210).

For the caspase-7 activity assay, MV-411-LAIR-1^overexpressing^ cells were treated as above for 4 days, followed by lysis with Pierce RIPA buffer (Thermo Fisher Scientific, 89901) supplemented with protease and phosphatase inhibitors. Total protein was quantified by Qubit protein BR assay (Invitrogen, A50668) following the manufacturer’s instructions. Equal amounts of protein from each sample were separated on a gradient gel, transferred to PVDF, blocked in 5% BSA, and probed with primary mAb or histone H3 antibodies (D11G5 and D1H2, respectively, Cell Signaling Technology). Images were taken by Azure 400 Blot Imager (Azure Biosystems). Data were quantified using ImageJ (NIH) software.

### NanoString nCounter and digital spatial imaging and quantification.

Protocols and reagents from NanoString were used for nCounter gene expression analysis and GeoMx digital spatial profiling (DSP).

For nCounter gene expression analysis, RNA was isolated from BM using Qiagen RNeasy kit, and differential gene expression was measured using the nCounter Metabolic Pathways Panel according to the manufacturer’s recommended protocol. Raw RCC files were analyzed using ROSALIND software. Counts were normalized to geometric means of housekeeping gene probes, and differential gene expression was calculated between isotype and treatment.

For DSP analysis, formalin-fixed, paraffin-embedded sections of tumor and spleen were deparaffinized and rehydrated, antigen-retrieved in citrate buffer, blocked, and stained with the Human Immune Profiling Core and Cell Death Panels overnight at 37°C. Anti–human CD45–Alexa Fluor 647 antibody (Novus Biologicals, NBP2-34528AF647) was used as a morphology marker at 5 μg/mL to identify MV4-11 cells. After washing, slides were fixed in 4% paraformaldehyde, and nuclei were stained with Syto13. Slides were scanned and regions of interest (human CD45^+^ regions) were acquired on a GeoMx DSP instrument (NanoString). Approximately 3–11 regions of interest were collected per tissue, and samples were multiplexed and processed on the nCounter Prep Station and Digital Analyzer as recommended. Data were analyzed on DSP Analysis software. Following quality control, data were normalized to housekeeping genes, and statistics were performed using Student’s *t* test.

### Statistics.

Statistical comparisons were made with GraphPad PRISM software using 2-tailed Student’s *t* test, 1-way ANOVA with multiple comparisons, or 2-way ANOVA as indicated in the figure legends. A *P* value less than 0.05 was considered significant. All data are shown as the mean ± SEM.

### Study approval.

All patients gave informed consent to participate in this study, which had the approval and guidance of the Institutional Review Board at Vanderbilt University Medical Center (VUMC). For PDX studies, all mouse procedures were performed in accordance with institutional guidelines at VUMC. Mice were maintained according to NIH animal care guidelines, and experimental protocols described in this study were approved by the VUMC Institutional Animal Care and Use Committee. CDX studies were performed at NextCure (Beltsville, Maryland) according to the *Guide for the Care and Use of Laboratory Animals* (37).

### Data availability.

Values for all data points in graphs are reported in the Supporting Data Values file. Data are available from the corresponding author upon request.

## Author contributions

RRL, DBF, and TKK wrote the manuscript. RRL, JH, SK, CMF, QH, KWK, HER, AEG, LSF, LT, AP, PK, IM, YM, NM, EFM, EAB, and AM performed experiments. JB, KM, and DBF constructed and characterized antibodies. RRL, LT, SJP, HM, MRS, DBF, and TKK conceived experiments and interpreted results. RRL, SK, SM, ZC, SJP, MRS, SL, HM, DBF, and TKK provided conceptual input. The order of the co–first authors was determined by giving placement priority to the individual responsible for writing the manuscript (RRL).

## Supplementary Material

Supplemental data

Supporting data values

## Version 1 11/15/2023

Electronic publication
