# Supplementary material for: LAIR-1 agonism as a therapy for acute myeloid leukemia
Source: J Clin Invest. 2023 Nov 15;133(22):e169519. doi: 10.1172/JCI169519 (PMC10650974; doi:10.1172/JCI169519)
Supplement: Supplemental data [file jci-133-169519-s009.pdf]

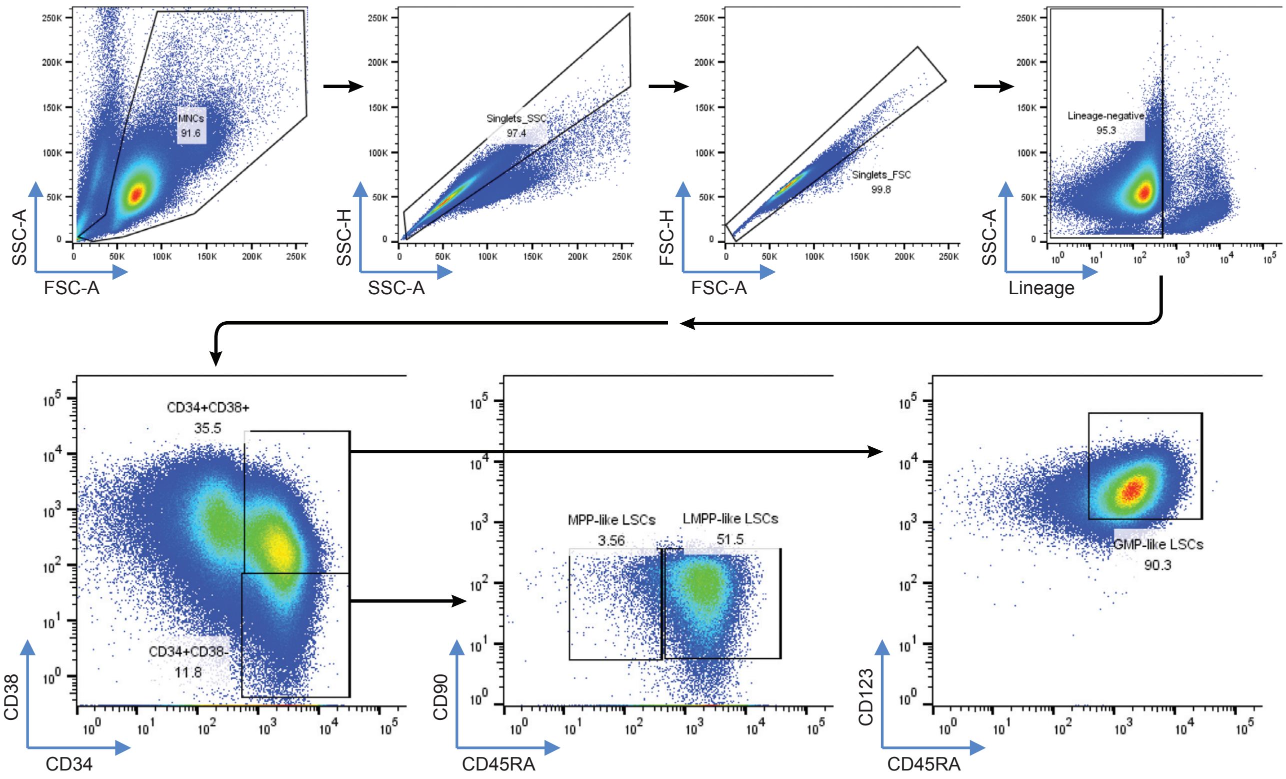

Supplemental Figure S1. LAIR-1 cell surface expression analysis. Gating schematic for quantification of LAIR-1 on primary patient BM cell subpopulations.

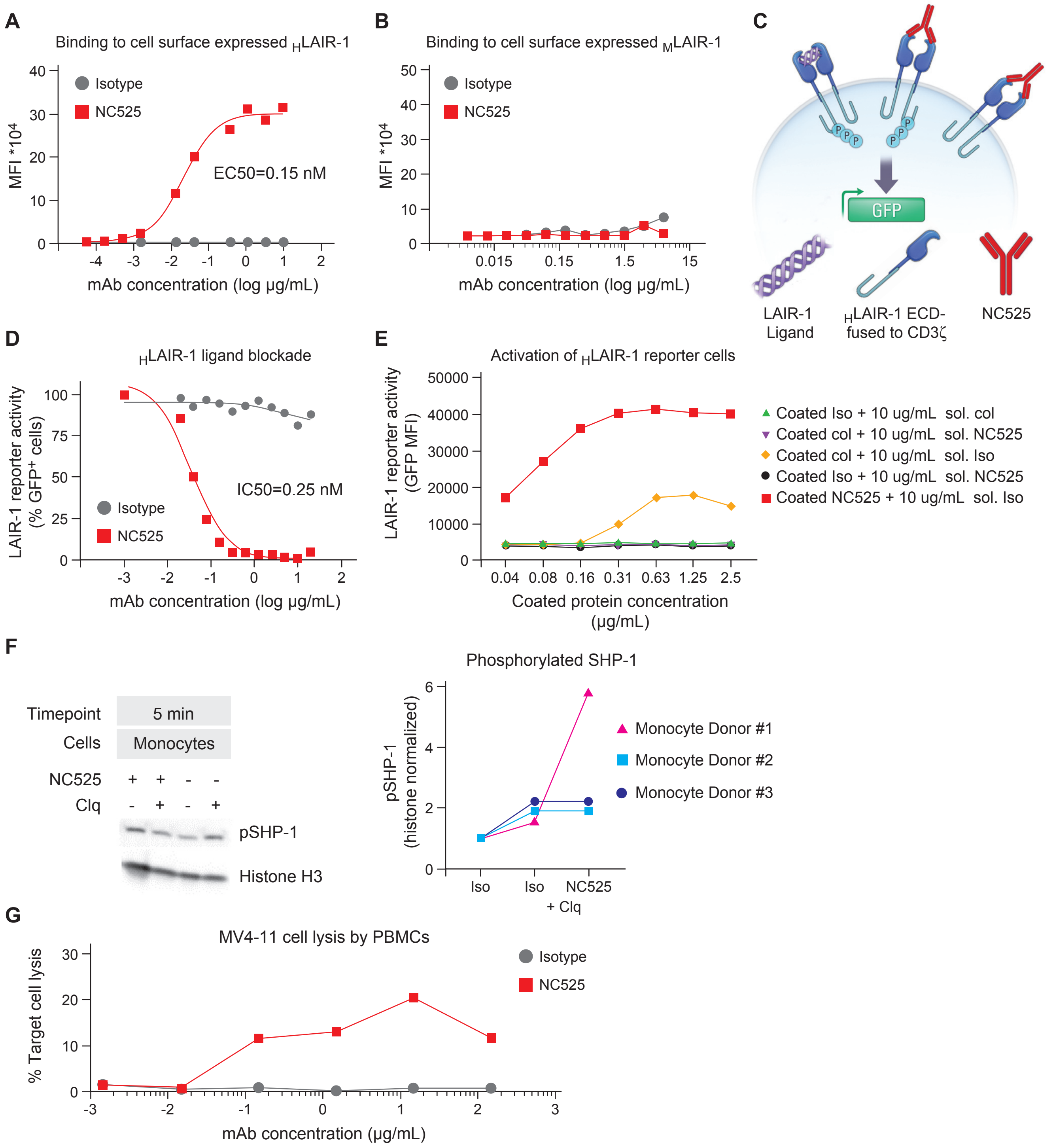

Supplemental Figure S2. Characterization of anti-LAIR-1 agonist monoclonal antibody NC525. (A) Binding profile of NC525 to human (H) LAIR-1^{+} cells or **(B)** mouse (M) LAIR-1^{+} cells. **(C)** Schematic of human LAIR-1 reporter cell line UT-140. UT-140 cells that express GFP under the NFAT promoter were transduced with human LAIR-1 fused to the zeta chain of CD3. Upon engagement of the LAIR-1 extracellular domain (ECD), signal transduction activates GFP fluorescence. **(D)** Profile of NC525 blockade of collagen-1 binding to LAIR-1, measured by UT-140 reporter cell activation. **(E)** Activation profiles of UT-140 LAIR-1 reporter cells by NC525, collagen, or isotype (Iso) under the indicated coated or soluble (sol) treatment conditions. **(F)** (left) Western blot and (right) quantification (pixel density normalized to histone H3) for phosphorylated SHP-1 in healthy donor blood monocytes under the indicated NC525, complement C1q, or isotype treatment conditions. Each line represents an individual donor. **(G)** MV4-11 cell lysis after coincubation with healthy donor peripheral blood mononuclear cells (PBMCs) at an 80:1 ratio in the presence of 10 µg/mL soluble NC525 or isotype control. Target cell lysis is plotted against mAb concentration. For graphs **AD, G**, isotype treatment is indicated by gray circles; NC525 treatment is indicated by red squares.

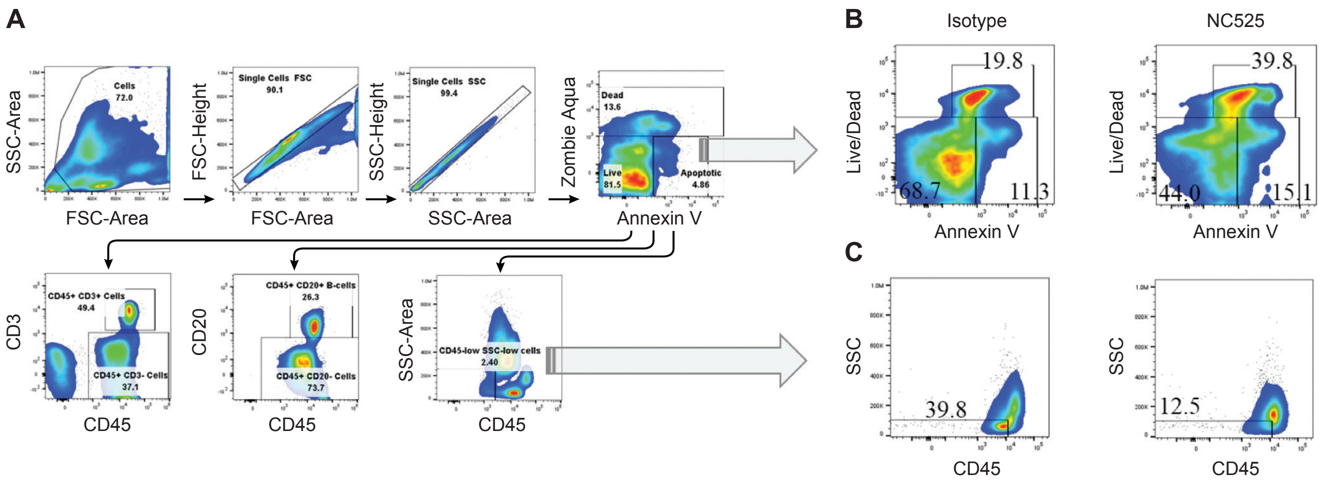

Supplemental Figure S3. Quantification of NC525-mediated AML cell death ex vivo. (A) Representative flow cytometry gating and scatterplots of **(B)** primary live, dead, or apoptotic total blood leukocytes or **(C)** $CD45^{Lo}$ SSC^{Lo} blast cells after ex vivo treatment with 10 ug/mL isotype control or NC525 in the presence of collagen.

Supplemental Figure S4

A

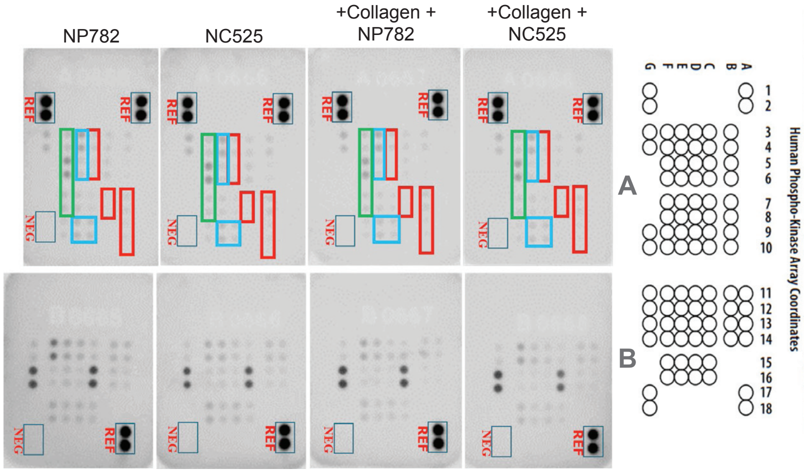

Membrane/ Coordinate	Target/Control	Phosphorylation Site
A-A1, A2	Reference Spot	——
B-A11, A12	Akt 1/2/3	T308
B-A13, A14	Akt 1/2/3	S473
B-A17, A18	Reference Spot	——
A-B3, B4	CREB	S133
A-B5, B6	EGF R	Y1086
A-B7, B8	eNOS	S1177
A-B9, B10	ERK1/2	T202/Y204, T185/Y187
B-B11, B12	Chk-2	T68
B-B13, B14	c-Jun	S63
A-C3, C4	Fgr	Y412
A-C5, C6	GSK-3α/β	S21/S9
A-C7, C8	GSK-3β	S9
A-C9, C10	HSP27	S78/S82
B-C11, C12	p53	S15
B-C13, C14	p53	S46
B-C15, C16	p53	S392
A-D3, D4	JNK 1/2/3	T183/Y185, T221/Y223
A-D5, D6	Lck	Y394
A-D7, D8	Lyn	Y397
A-D9, D10	MSK1/2	S376/S360
B-D11, D12	p70 S6 Kinase	T389
B-D13, D14	p70 S6 Kinase	T421/S424
B-D15, D16	PRAS40	T246

Membrane/ Coordinate	Target/Control	Phosphorylation Site
A-E3, E4	p38α	T180/Y182
A-E5, E6	PDGF Rβ	Y751
A-E7, E8	PLC-γ1	Y783
A-E9, E10	Src	Y419
B-E11, E12	PYK2	Y402
B-E13, E14	RSK1/2	S221/S227
B-E15, E16	RSK1/2/3	S380/S386/S377
A-F3, F4	STAT2	Y689
A-F5, F6	STAT5a/b	Y694/Y699
A-F7, F8	WNK1	T60
A-F9, F10	Yes	Y426
B-F11, F12	STAT1	Y701
B-F13, F14	STAT3	Y705
B-F15, F16	STAT3	S727
A-G1, G2	Reference Spot	——
A-G3, G4	β-Catenin	——
A-G9, G10	PBS (Negative Control)	——
B-G11, G12	STAT6	Y641
B-G13, G14	HSP60	——
B-G17, G18	PBS (Negative Control)	——

B

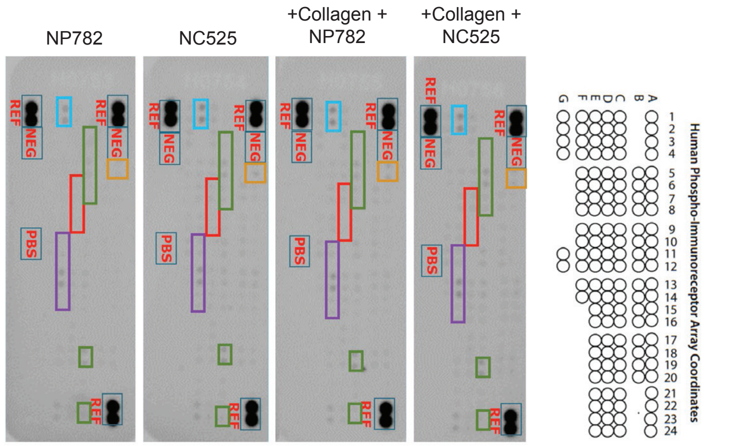

Coordinate	Receptor
A1, A2	Reference Spots
A3, A4	Bt-Control*
A5, A6	2B4/SLAMF4
A7, A8	BLAME/SLAMF8
A9, A10	BTLA
A11, A12	CD3ε
A13, A14	CD5
A15, A16	CD6
A17, A18	CD28
A19, A20	CD84/SLAMF5
A21, A22	Bt-Control*
A23, A24	Reference Spots
B5, B6	CD229/SLAMF3
B7, B8	CEACAM-1
B9, B10	CLEC-1
B11, B12	CLEC-2
B13, B14	CRACC/SLAMF7
B15, B16	CTLA-4/CD152
B17, B18	DCIR/CLEC4A
B19, B20	Dectin-1/CLEC7A
C1, C2	DNAM-1
C3, C4	Fcε RII/CD23
C5, C6	Fcγ RIIA
C7, C8	Fcγ RIIIA/B
C9, C10	FcRH1/IRTA5
C11, C12	FcRH2/IRTA4
C13, C14	FcRH4/IRTA1
C15, C16	FcRH5/IRTA2
C17, C18	ILT2/CD85j
C19, C20	ILT3/CD85k
C21, C22	ILT4/CD85d
C23, C24	ILT5/CD85a

*Biotinylated Control

Coordinate	Receptor
D1, D2	ILT6/CD85e
D3, D4	Integrin β3/CD61
D5, D6	KIR2DL4
D7, D8	LAIR-1
D9, D10	LAIR-2
D11, D12	LMIR1/CD300A
D13, D14	LMIR2/CD300C
D15, D16	LMIR3/CD300LF
D17, D18	LMIR6/CD300LE
D19, D20	MDL-1/CLEC5A
D21, D22	NKp30/NCR3
D23, D24	NKp44/NCR2
E1, E2	NKp46/NCR1
E3, E4	NKp80/KLRF1
E5, E6	NTB-A/SLAMF6
E7, E8	PD-1
E9, E10	PECAM/CD31
E11, E12	SHIP-1
E13, E14	SHP-1
E15, E16	SHP-2
E17, E18	Siglec-2/CD22
E19, E20	Siglec-3/CD33
E21, E22	Siglec-5
E23, E24	Siglec-7
F1, F2	Siglec-9
F3, F4	Siglec-10
F5, F6	SIRP-β1
F7, F8	SLAM/CD150
F9, F10	TREM-1
F11, F12	TREM-2
F13, F14	TREML1/TLT-1
G1, G2	Reference Spots
G3, G4	Bt-Control*
G11, G12	PBS

Supplemental Figure S4. NC525 and collagen induced phosphorylation signaling. (A) Human phosphokinase array dot blots and **(B)** human phospho-immunoreceptor array dot blots with the respective keys for AML patient PBMCs treated with 10 µg/mL isotype control, LAIR-1 agonist mAb NC525, 50 µg/mL collagen-1 plus isotype control, or collagen-1 plus NC525.

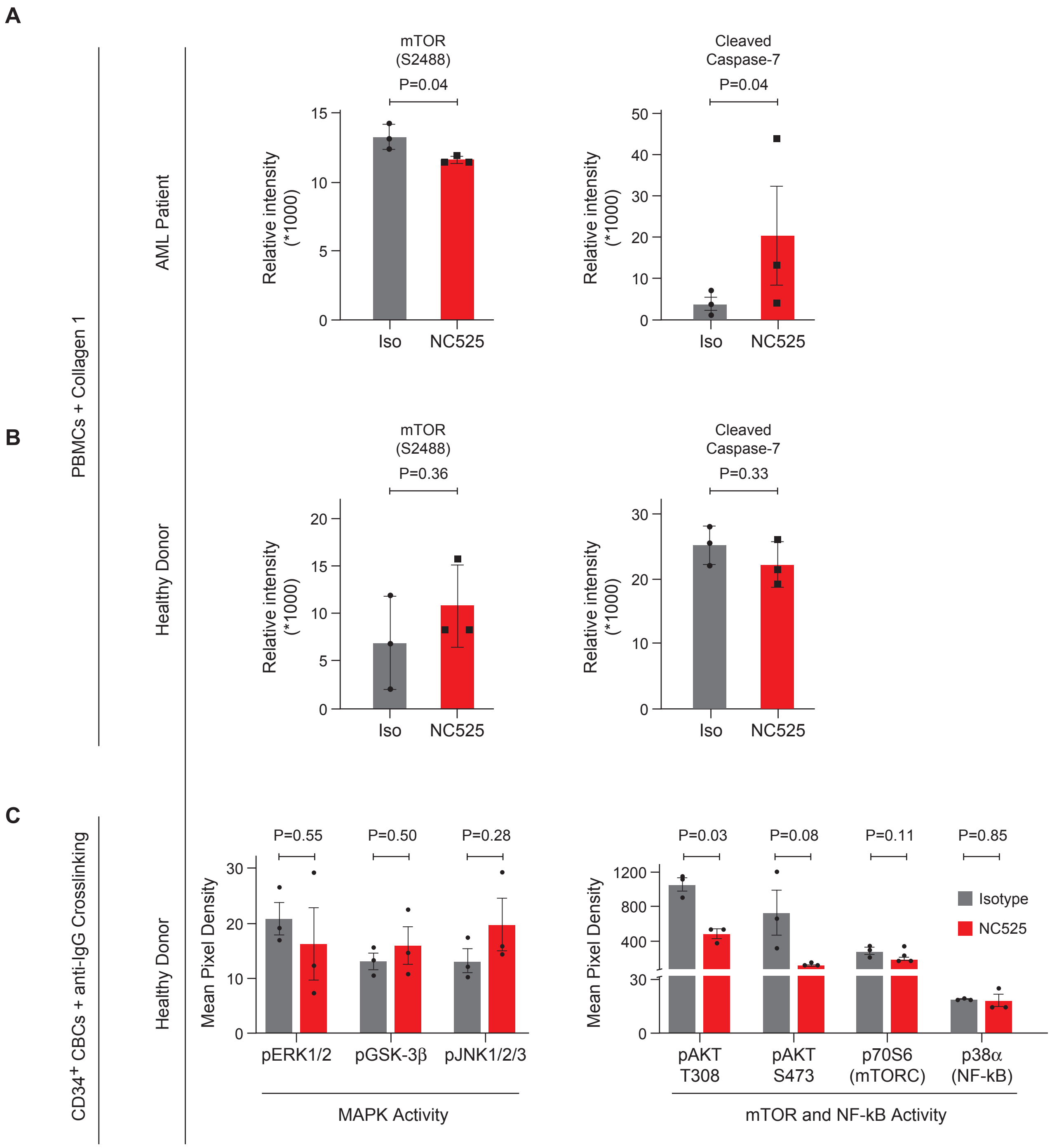

Supplemental Figure S5. LAIR-1 mAb NC525 does not induce cytotoxic signaling in healthy cells. Reverse phase protein microarray of mTOR or caspase-7 activation in **(A)** AML donor or **(B)** healthy donor PBMCs cultured in 50 ug/mL coated collagen-1 and treated with 10 ug/mL isotype (gray) or NC525 (red) for 5 minutes. **(C)** Immunophosphoarray of $CD34^+$ enriched cord blood cells (CBCs) treated with 10 ug/mL isotype (gray) or NC525 (red) and anti-IgG for 30 minutes. N = 3 technical replicates per condition. Error bars represent standard error of mean. P values determined by Student's T test.

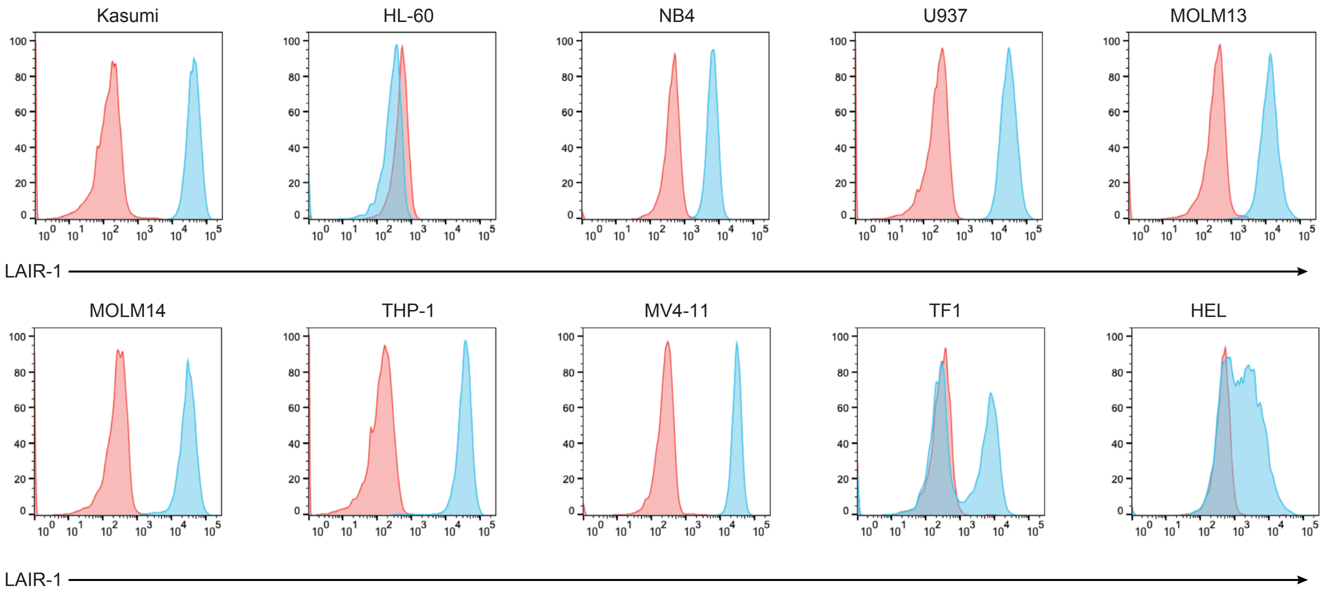

Supplemental Figure S6. LAIR-1 expression on AML cell lines. Histograms of LAIR-1 cell surface expression (blue) on the indicated AML cell lines relative to isotype control staining (red).

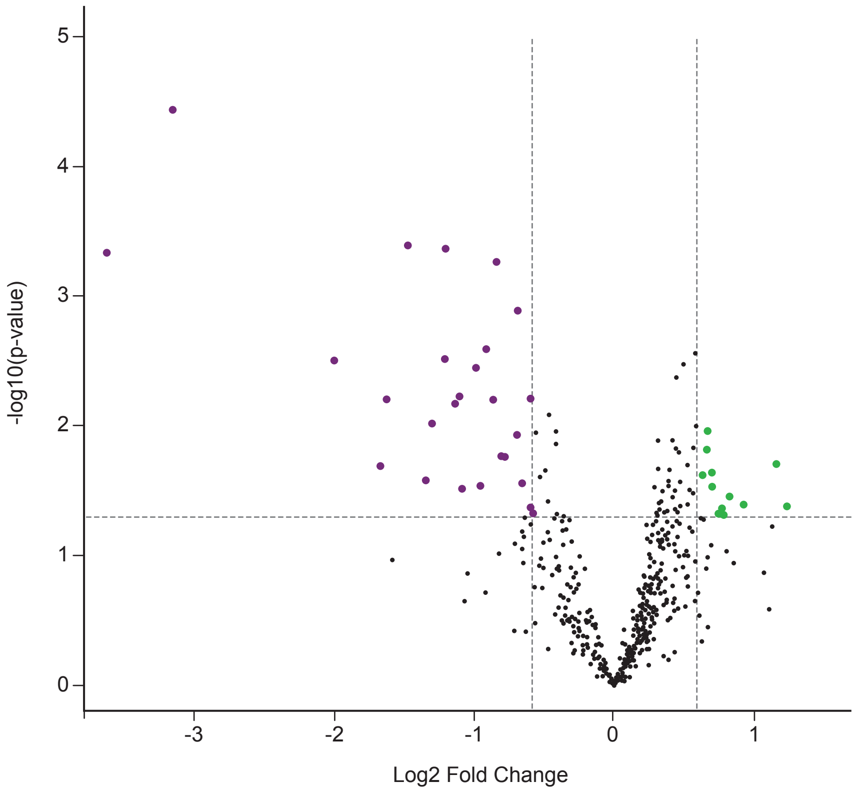

Supplemental Figure S7 Differential gene expression in CDX mouse bone marrow. Significantly downregulated (purple) or upregulated (green) genes in MV4-11 cells recovered at day 27 postchallenge from the bone marrow of CDX mice treated with NC525 compared to isotype control. Significance threshold set at P value < 0.05. Gene values and descriptions are listed in Supplemental Table S3.

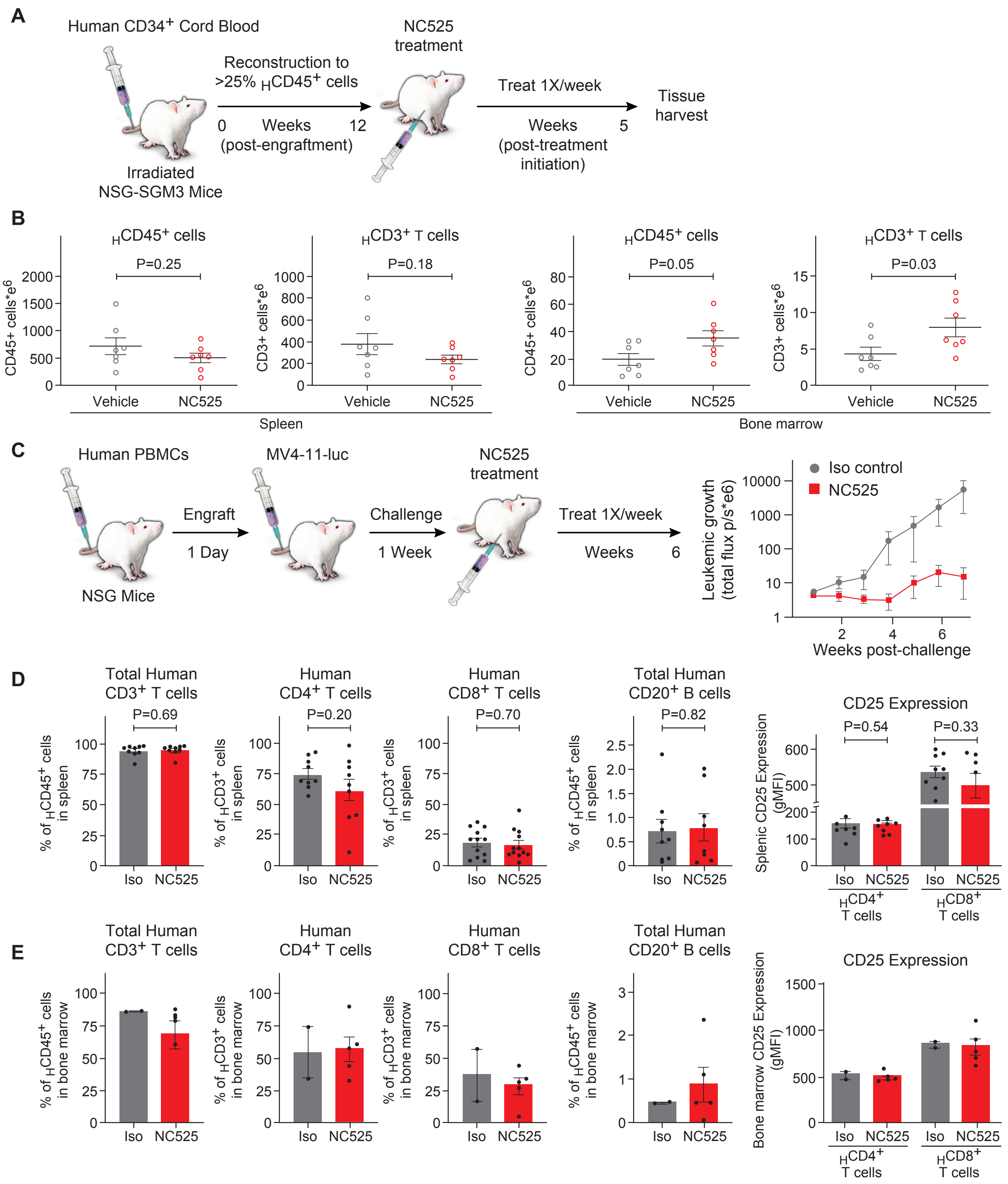

Supplemental Figure S8. LAIR-1 monoclonal antibody NC525 does not impact healthy leukocytes. **(A)** Schematic of the model system for defining NC525 effects on human ($_H$) immune cells in vivo. **(B)** Cell counts of human CD45$^+$ cells or human CD3$^+$ cells in the spleen or bone marrow of engrafted mice treated with vehicle (gray) or 10 mg/kg NC525. N = 7 mice per group. P values determined by Student's T test. **(C)** Model schematic and leukemic growth of MV4-11 challenged mice engrafted with human PBMCs. N = 68 mice. **(D)** Percent of human CD3$^+$ T cells, CD4$^+$ T cells, CD8$^+$ T cells, CD20$^+$ B cells, and the degree of CD25 expression on CD3$^+$ T cells recovered from the spleen or **(E)** bone marrow of PBMC engrafted CDX mice after treatment with 10 mg/kg isotype (gray) or NC525 (red). Bar graphs are generated from mice euthanized at day 28 postchallenge. N = 29 biological replicates. Error bars represent standard error of mean. P values determined by Student's T test.

	Types of AML	BM blast %	Karyotype	NGS data
AML01	NK	89.5	46,XX[20]	Not done
AML02	FLT3 mutated	97.5	46,XY,t(1;4)(p36.1;q31)[7]/46,idem,del(7)(q34)[3]/48,XY,+8,+13[10]7	FLT3 ITD p.Val581_Phe594dup (47%)<br>IDH2 p.Arg172Lys (96%)
AML03	CK without monosomy	97	47,XX,-8,-13,?del(18)(q21),+21,+2mar[5]/46,XX,add(21)(q22)[2]/46,XX[14]	FLT3 ITD p.Arg595_Leu601dup, and splice site variant, p.?. WT1 : p.Ala170tyrfs*70 and p.Ser169*
AML04	t(8;21)	62	46,XX,t(8;21)(q22;q22)[20]	Tier II: Variants of Potential Clinical Significance<br>EZH2 p.Arg63Profs*10 (10%)<br>Tier III: Variants of Unknown Clinical Significance<br>EZH2 p.Gly743_Ile744insGlyGly (7%)<br>RUNX1 p.Ser172Asn (11%)
AML05	inv(16)	63	46,XY,der(7)t(7;16)(q22;q22)inv(16)(p13.1q22),der(16)t(7;16)inv(16)(p13.1q22)[18]/46,XY[2]	NGS not done.<br>AML Snapshot Panel: No mutations detected in IDH1, IDH2, FLT-3 (TKD), KIT<br>No mutations detected in FLT3, NPM1, BEBPA
AML06	TP53 mutated	31.5	44,XX,del(5)(q22q35),add(12)(p13),-13,add(13)(p11.2),-16[cp9]/48,idem,+X,+8,add(13)(p11.2),+22[10]/46,XX[1]	TP53 p.Cys242Ser (90%)
AML07	FLT3 mutated	77	46,XY,+1,der(1;15)(q10;q10)[9]/43~46,XY,i(1)(q10)[cp2]/46,XY[9]	CSF3R p.Thr618Ile (38%)<br>FLT3-TKD p.Asp835Tyr (43%)<br>SRSF2 p.Pro95Arg (50%)<br>TET2 p.Leu1332_Met1333delinsProLeu(45%)<br>ZRSR2 p.Arg126* (81%)
AML08	Monocytic leukemia	87.5	46,XX[20]	NGS not done.<br>No mutations found in FLT3, CEBPA, cKIT
AML09	t(8;21)	35	45,X,-Y,t(8;21)(q22;q22)[5]/45,idem,del(9)(q21q22)[9]/46,XY[6]	One insertion mutation in KIT(p.Tyr418_Asp419insPhePhe) was identified in this patient's sample
AML10	inv(16)	83.5	46,XX,del(7)(q22),del(9)(p22),del(12)(p12),inv(16)(p13.1q22)[cp20]	NGS not done.<br>No mutations found in FLT3, NPM1, CEBPA. cKIT positive.
AML11	NK	94	46,XY[20]	NGS not done. No mutations in IDH1/2, FLT3-TKD, DNMT3A or KIT. FLT3-ITD positive mutant: wild type ratio is 0.15.
AML12	inv(16)	90	46,XX,inv(16)(p13.1q22)[17]/46,XX[3]	NGS not done.<br>Negative for mutations in FLT3, NPM1, CEBPA, cKIT
AML13	t(8;21)	70	45,X,-X,t(8;21)(q22;q22)[19]/46,XX[1]	NGS not done.<br>Negative for mutations in FLT3, NPM1, CEBPA.<br>KIT (c.2446G>A and c.2447A>T) D816I (GAC>ATC)DETECTED<br>The presence of the double mutation at c.2446 and c.2447 in KIT was confirmed by sequencing.
AML14	CK with monosomy	48.5	43~46,XY,add(2)(q31),add(3)(p21),add(4)(q21),del(5)(q15q33),-7,+8,-12,add(13)(q34),add(15)(p11.2),- 16,-18,+1~2mar[cp20]	TP53 p.Leu114Cysfs*9 (44.9%) and p.? (40.6%)
AML15	CK without monosomy	88.5	46~49,XY,+8,t(8;16)(p11.2;p13.3),add(22)(p11.2),+mar[cp18]/46,XY[2]	TET2 (c.5081T>A; p.Leu1694*; 41%)<br>TET2 (c.1936dup; p.Thr646Asnfs*35; 43%)<br>VUS in ASXL1 (c.3538G>C; p.Asp1180His; 49%)
AML16	Monocytic leukemia	89	46,XX[20]	NGS not done.<br>Negative for mutations in FLT3, NPM1, CEBPA, cKIT
AML17	CK with monosomy	70	40-42,XX,add(3)(q12),add(5)(q11.2),-7,-9,-12,add(16)(q12),-17,add(18)(q21),19,-20,-22,+1-2mar[cp20]	Not performed
AML18	TP53 mutated	81	46,XY,t(11;19)(q23;p13.1)[9]/47,idem,+mar[11]	TP53 p.Arg248Trp (45%)<br>Tier II: NRAS p.Gly12Cys (88%)
AML19	NK	70	46,XX[20]	NGS not done.<br>IDH2 mutation detected. No mutations detected in FLT3, NPM1, CEBPS
AML20	NK	79	46,XX[20]	NPM1 p.Trp288Cysfs*12 (38.6%)<br>BCOR p.Cys1606Asnfs*11 (40.2%)<br>NRAS p.Gly13Asp (VAF 43.2%)
AML21	TP53 mutated	90	42~46,XY,-Y,+8,der(9)t(9;11)(q34;q12),der(12;17)(q10;q10),der(17)t(3;17)(q13;p12)[cp20]	TP53 p.Cys275Tyr (77%) and TP53 p.Thr102Profs*21 (11%)
AML22	TP53 mutated	71.5	45~47,XX,-5,add(7)(q11.2),+8,+11,der(11)t(11;11)(p15;q13),add(16)(q21),psu did(17;5)(p11.2;q22),-17,-20,+1~2mar[cp16]/46,XX[4]	TP53 p.Phe113Val (45%)
AML23	Monocytic leukemia	48.5	46,XY[20]	NGS not done.<br>Negative for cKIT.
AML24	Monocytic leukemia	40	46,XX[20]	NGS not done.
AML25	Monocytic leukemia	54.3	45,XY,inv(3)(q21q26.2),-7[17]/45,idem,del(5)(q22q35)[3]	NGS not done.<br>Positive for cKIT mutation. Negative for mutations in FLT3, NPM1, CEBPA, BCR/ABL1

Supplemental Table S1. AML patient samples. Summary and description of AML patient BM samples tested for LAIR-1 expression. Headings denote patient/sample AML type, percent circulating blasts, karyotype, and molecular genotype as determined by sequencing (NGS).

Assay	Patient #	Types of AML	BM blast %	Karyotype	NGS data	Previous treatment
CFU	1	t(8;21)	35	45,X,-Y,t(8;21)(q22;q22)[5]/45,idem,del(9)(q21q22)[9]/46,XY[6]	One insertion mutation in KIT(p.Tyr418_Asp419insPhePhe) was identified in this patient's sample	None
CFU	2	NK	94	46,XY[20]	NGS not done. No mutations in IDH1/2, FLT3-TKD, DNMT3A or KIT. FLT3-ITD positive mutant: wild type ratio is 0.15.	Yes: Status post low dose ARA-C, s/p 3+7 induction chemotherapy
CFU	3	inv(16)	90	46,XX,inv(16)(p13.1q22)[17]/46,XX[3]	NGS not done. Negative for mutations in FLT3, NPM1, CEBPA, cKIT	None
CFU & PDX	4	t(8;21)	84.5	47,XX,+13[16]/48,idem,+13[1]/46,XX[3]	ASXL1 p.Glu635Argfs*15 (40%) BCOR p.Glu1076Glyfs*3 (37%) RUNX1 p.Ser388* (85%) SRSF2 p,Pro95His (48%)	None
CFU	5	t(8;21)	90	46,XX,add(1)(p36.1),t(8;21)(q22;q22)[20]	NSG not done	status post 7+3 induction with persistent disease, 5+2 reinduction with CR1, HiDAC x4, with relapse 4/2018, s/p FLAG-M with CR2 and 2 cycles consolidation
CFU	6	APL	64.5	46,XX,t(15;17)(q24;q21),add(17)(p11.2)[19]/46,XX[1]	NGS not done	None
CFU	7	NK	92	47,XY,+mar[2]/46,XY[18]	NGS not done	None
CFU	8	AML-MRC	28	45,XY,del(5)(q22q35),-16,add(17)(p11.2)[10]/45,idem,inv(7)(p15q11.2)[9]/46,XY[1]	NGS not done	None
PDX	9	NK	N/A	N/A	NGS not done	N/A
PDX	10	AMML	N/A	N/A	NGS not done	N/A
PDX	11	Monocytic FLT3^{+}	<5	Abnormal	EZH2chr7:148506443, c.2069G>A, p.Arg690His; NM_004456.4Missense39.9FLT3chr13:28592640, c.2505T>G, p.Asp835Glu abnormal clone with an extra copy of chromosomes 6 and 13, and a translocation between chromosomal bands 11q23 and 17q25 Acquired t(11;17)(q23;q25), likely with underlying KMT2A/SEPT9 gene fusions	None
PDX	12	FLT-3+, ITD	N/A	N/A	NPM1 chr5:170837543 c.859_860insTCTG p.Trp288fs NM_002520.6 Frameshift Insertion 53.4	None

Supplemental Table S2. AML patient samples. Summary and description of AML patient BM utilized in CFU and PDX modeling. Headings denote the assay(s) which utilized the respective patient/sample, patient and sample identifiers (ID), AML type, percent circulating blasts, karyotype, and molecular genotype as determined by sequencing (NGS) and treatment history if available.

Sample #	Assay	Primary Diagnosis	Sex	Race	Age	Previous treatments	Current Medications
**122042476**	Whole Blood Cell Killing	AML	M	Black	66	Daunorubicin/Cytarabine (7 + 3 induction), Mylotarg x1 with HiDAC x 2	acyclovir, Colace, Vitamin B12, Duloxetine, isosorbide mononitrate, nystatin, Nystatin/ Benadryl/Lidocaine /Mylanta, ondansetron, posaconazole, promethazine
**122121088**	Whole Blood Cell Killing	AML	F	American Indian	80	N/A	Dacogen/Venclexta
**121982560**	Whole Blood Cell Killing	AML	M	White	61	N/A	Dacogen
**311906**	Whole Blood Cell Killing	AML (M1)	F	White	62	Induction with 7+3 chemotherapy (3/19 – 4/19); recovery BM (4/19) showed refractory disease; Tolero Clinical Trial Therapy (4/23/19-7/15/19); HiDAC (9/16/19); IDAC (12/3/19). Repeat bone marrow biopsy (12/30/2020) showed AML relapse; Venetoclax/Decitabine x 7 cycles (last 10/9/21); Restaging BM 11/17/21 showed morphologic remission with detectable 1q duplication. Pt relapsed (3/30/22) while undergoing work up for SCT	N/A
**121769827**	Whole Blood Cell Killing	AML	M	White	75	Elitek	Venetoclex/DEX
**200013115**	Signaling (phosphoarray)	AML	M	N/A	74	Newly diagnosed	Newly diagnosed
**2002170121**	Signaling (reverse phase microarray)	AML	M	White	64	Cytoxan	N/A
**16761**	Bone Marrow Cell Killing	AML	Female	N/A	63	1st line Idarubicin (Cytarabine Cyt); 2nd line fludarabine, Cyt, Ida, G-CSF; 3rd line Venetoclax, Azacitidine	N/A

Supplemental Table S3. Patient samples used in ex vivo whole blood killing assays or PBMC signaling studies. Headings denote the assay which utilized the respective sample, patient diagnosis, sex, race, age, and treatment history (if available).
N/A = not available/unknown.

Cell Line	Cell Type	Morphology	FAB Classification	Molecular Mutation	Disease	LAIR-1 Expression
**MV4-11**	Macrophage	Lymphoblast	M5	FLT3 ITD+	Biphenotypic B Myelomonocytic Leukemia	++
**THP-1**	Monocyte	Monocyte	M5	NA	Acute monocytic leukemia	++
**Kasumi**	Myeloblast	Myeloblast	M2	RUNX1-RUNX1T1	Acute myeloblastic leukemia	++
**HL-60**	Promyeloblast	Lymphoblast-like	M3	PML-RARa	Acute promyelocytic leukemia	-
**NB-4**	Myeloblast	Myeloblast	M3	PML-RARa	Acute promyelocytic leukemia	+
**MOLM-13**	Monocyte	Lymphoblast-like	M5	FLT3 ITD+	Acute myeloid leukemia	++
**MOLM-14**	Monocyte	Lymphoblast-like	M5	FLT3 ITD+	Acute myeloid leukemia	++
**U-937**	Monocyte	Monocyte	M4/5	NA	Histiocytic Lymphoma	++
**TF-1**	Erythroblast	Lymphoblast	M6	NA	Erythroleukemia	+
**HEL**	Erythroblast	Lymphoblast	M6	NA	Erythroleukemia	+

Supplemental Table S4. Leukemic cell lines tested for LAIR1 expression. Summary and description of AML cell lines tested for LAIR-1 expression. Headings denote cell type, morphology, FAB classification, molecular mutation, and disease diagnosis of source patient. NA = not applicable/unknown.

		Isotype Control Treated Mice			NC525 Treated Mice						
Symbol	Description	Mouse # SCL0342	Mouse # SCL0343	Mouse # SCL0344	Mouse # SCL0346	Mouse # SCL0347	Mouse # SCL0348	log2FoldChange	Fold Change	pvalue	padj
SLC27A1	solute carrier family 27 (fatty acid transporter)_ member 1	6.559366885	5.215477085	5.589872352	5.583309685	5.196492224	4.925035976	-3.614709844	12.25	0.000465733	0.053012861
MYCL	v-myc avian myelocytomatosis viral oncogene lung carcinoma derived homolog	5.833541848	5.834386917	5.589872352	5.583309685	5.196492224	4.995425304	-3.14838375	8.866616947	3.60E-05	0.017240274
NFAT5	nuclear factor of activated T-cells 5_ tonicity-responsive	5.887989632	4.867553781	5.6283465	5.080809344	5.476600144	4.851035395	-2	4	0.003175944	0.143048032
RIMKLB	ribosomal modification protein rimK-like family member B	6.624955227	5.693524382	5.187773909	5.955278462	5.637064816	4.690570722	-1.669851398	3.181818182	0.020656085	0.265292287
NAT8L	N-acetyltransferase 8-like (GCN5-related_ putative)	6.525419553	6.51664662	5.509702004	5.888164266	5.476600144	5.690570722	-1.624490865	3.083333333	0.006222179	0.177557019
ASH1L	ash1 (absent_ small_ or homeotic)-like (Drosophila)	10.06088009	9.774444377	8.872272083	8.035005654	8.422560304	8.410462803	-1.475926855	2.781622912	0.000408896	0.053012861
ACSF3	acyl-CoA synthetase family member 3	5.77695832	5.693524382	5.187773909	5.888164266	5.296027898	4.995425304	-1.345774837	2.541666667	0.026527833	0.279132051
ZNF708	zinc finger protein 708	8.144329386	9.444295775	8.194200178	7.110556687	7.764176734	7.869894422	-1.302078319	2.465838509	0.00956873	0.224918328
MTOR	mechanistic target of rapamycin (serine/threonine kinase)	9.445985086	9.615136483	8.488943444	7.888164266	7.896931943	8.614335136	-1.208787124	2.311432326	0.003081932	0.143048032
SOS1	son of sevenless homolog 1 (Drosophila)	8.901286455	8.958868948	8.30523149	7.495846843	7.896931943	7.732390898	-1.208560148	2.311068702	0.000434027	0.053012861
PLCG1	phospholipase C_ gamma 1	7.455030225	6.851065659	6.4027868	6.080809344	6.389137302	6.48575093	-1.133583154	2.194029851	0.006731035	0.179120312
PRR5	proline rich 5 (renal)	6.656664086	6.915916803	6.238399982	6.250734345	5.848568921	6.188070382	-1.10433666	2.15	0.005940685	0.177557019
EME1	essential meiotic structure-specific endonuclease 1	8.28284925	7.008035005	6.72026899	6.705300209	7.117057757	6.357995383	-1.08979667	2.128440367	0.030843097	0.279132051
D2HGDH	D-2-hydroxyglutarate dehydrogenase	8.464019008	8.447041152	7.823362483	7.328736857	7.728987305	7.331028336	-0.9881259	1.983606557	0.003583667	0.143048032
HERC1	HECT and RLD domain containing E3 ubiquitin protein ligase family member 1	10.70864138	9.169673395	9.184550008	9.019408799	9.003847146	8.929537368	-0.960670153	1.94621373	0.029187867	0.279132051
INSR	insulin receptor	9.884646189	9.399644009	8.880264874	8.583309685	8.366417226	8.851035395	-0.914352905	1.884723524	0.002570416	0.143048032
FANCA	Fanconi anemia_ complementation group A	9.87119439	10.92677532	9.866248296	9.408736179	9.533795545	9.515997217	-0.867597141	1.824621397	0.006301606	0.177557019
BCL2	B-cell CLL/lymphoma 2	8.791313613	8.495585004	8.488943444	7.743774357	7.655923843	8.210438195	-0.838693452	1.788429752	0.00055337	0.053012861
RANBP2	RAN binding protein 2	10.04599147	9.115481295	8.768436272	8.540240963	8.728987305	8.768014808	-0.80479013	1.746891652	0.017167933	0.245310254
UMPS	uridine monophosphate synthetase	6.262385147	6.3388595	6.823362483	6.250734345	6.247118297	6.029372636	-0.780882711	1.718181818	0.017412419	0.245310254
CSF3R	colony stimulating factor 3 receptor (granulocyte)	9.644854784	10.15463648	9.340420919	9.011546682	9.170497016	9.134489342	-0.695556525	1.619509044	0.011738952	0.224918328
HJURP	Holliday junction recognition protein	9.098886415	8.825974678	8.56529433	8.035005654	8.354921587	8.32420707	-0.69503973	1.618929016	0.001289798	0.102968855
KAT6A	K(lysine) acetyltransferase 6A	10.67806656	9.867553781	9.735770083	9.203205975	9.806989817	9.527920385	-0.659803728	1.579867675	0.027556069	0.279132051
NCOA2	nuclear receptor coactivator 2	7.490654135	7.5679935	7.711335865	6.705300209	7.271780352	7.384467595	-0.639689428	1.55799373	0.051276919	0.29232424
BRCA2	breast cancer 2_ early onset	8.499424345	8.332934666	8.148603312	7.6043713	8.061562644	7.89772863	-0.600441485	1.516180468	0.00613197	0.177557019
ACACA	acetyl-CoA carboxylase alpha	6.656664086	6.051978353	6.311156324	6.402737439	5.848568921	6.217817725	-0.598137889	1.513761468	0.042950771	0.29232424
ATF7IP	activating transcription factor 7 interacting protein	9.477398038	8.302939926	8.451881349	8.264031168	8.366417226	8.357995383	-0.596128927	1.511655012	0.057612365	0.29232424
PTGER4	prostaglandin E receptor 4 (subtype EP4)	6.991083125	5.765674167	6.311156324	6.450043154	6.196492224	6.0625395	-0.584962501	1.5	0.048328804	0.29232424
CYBB	cytochrome b-245_ beta polypeptide	8.381029644	8.108561881	8.451881349	9.390664607	8.366417226	8.951836035	0.661753754	0.632109431	0.015415536	0.238194896
NDUFB2	NADH dehydrogenase (ubiquinone) 1 beta subcomplex_ 2_ 8kDa	10.61288239	10.56673185	10.77488168	11.83458323	10.92441268	11.0061199	0.666378997	0.63008615	0.010932197	0.224918328
APRT	adenine phosphoribosyltransferase	10.76063601	10.35063667	10.65188201	11.85339885	10.90680669	10.86989442	0.692839902	0.618634886	0.022959069	0.268229127
LTA4H	leukotriene A4 hydrolase	6.303027132	6.765674167	7.1617787	7.561936034	7.247118297	7.410462803	0.698209551	0.616336634	0.02962039	0.279132051
PKM	pyruvate kinase_ muscle	10.72555917	10.3210115	10.47317613	12.02917658	10.45253238	10.76801481	0.743042077	0.597478177	0.047862354	0.29232424
GABARAP	GABA(A) receptor-associated protein	11.12886928	11.14113748	11.61942074	12.58330968	11.56655684	11.88735363	0.764850103	0.588514512	0.043275492	0.29232424
PRKAB2	protein kinase_ AMP-activated_ beta 2 non-catalytic subunit	5.77695832	4.993084664	6.108339441	6.353827838	6.247118297	6.246964071	0.779231321	0.582677165	0.048356315	0.29232424
NDUFB11	NADH dehydrogenase (ubiquinone) 1 beta subcomplex_ 11_ 17.3kDa	9.894653363	9.468818094	9.768436272	11.30962803	9.768515669	9.997570576	0.819329649	0.566705202	0.035527504	0.29232424
SCD	stearoyl-CoA desaturase (delta-9-desaturase)	6.940457052	6.656049676	6.755458418	7.665771845	7.433531422	7.669197072	0.916858765	0.529661017	0.040896249	0.29232424
CD63	CD63 molecule	12.5135632	12.11935893	12.25808656	14.1206423	12.34696486	12.49868316	0.925316005	0.526565167	0.040558201	0.29232424
CDA	cytidine deaminase	5.991083125	5.730050258	5.6283465	7.110556687	5.848568921	6.625475694	1.154577037	0.449197861	0.019628932	0.265292287
ARF5	ADP-ribosylation factor 5	9.542493066	8.935599169	9.070416958	11.32398333	9.637064816	9.482691131	1.230535366	0.426159274	0.041758752	0.29232424

Supplemental Table S5. Significantly differential gene expression in CDX mouse bone marrow after NC525 treatment. Significantly different gene expression in MV4-11 cells recovered at day 27 post-challenge from the bone marrow of CDX mice. p-values and log2 fold changes, with an adjusted p-value < 0.05 and absolute log2 fold change > 1, define differentially expressed genes between 3 isotype-treated verses 3 NC525-treated CDX mice.
